# Development and validation of a prediction model for early mortality after transcatheter aortic valve implantation (TAVI) based on the Netherlands Heart Registration (NHR): The TAVI‐NHR risk model

**DOI:** 10.1002/ccd.30398

**Published:** 2022-09-07

**Authors:** Hatem Al‐Farra, Anita C. J. Ravelli, José P. S. Henriques, Saskia Houterman, Bas A. J. M. de Mol, Ameen Abu‐Hanna, NHR THI Registration Committee

**Affiliations:** ^1^ Department of Medical Informatics, Amsterdam UMC Location University of Amsterdam Amsterdam The Netherlands; ^2^ Heart Centre, Amsterdam Cardiovascular Sciences, Amsterdam UMC Location University of Amsterdam Amsterdam The Netherlands; ^3^ Amsterdam Public Health Amsterdam The Netherlands; ^4^ Netherlands Heart Registration Utrecht The Netherlands

**Keywords:** aortic stenosis, internal validation, mortality, prediction model, transcatheter aortic valve implantation (TAVI)

## Abstract

**Background:**

The currently available mortality prediction models (MPM) have suboptimal performance when predicting early mortality (30‐days) following transcatheter aortic valve implantation (TAVI) on various external populations. We developed and validated a new TAVI‐MPM based on a large number of predictors with recent data from a national heart registry.

**Methods:**

We included all TAVI‐patients treated in the Netherlands between 2013 and 2018, from the Netherlands Heart Registration. We used logistic‐regression analysis based on the Akaike Information Criterion for variable selection. We multiply imputed missing values, but excluded variables with >30% missing values. For internal validation, we used ten‐fold cross‐validation. For temporal (prospective) validation, we used the 2018‐data set for testing. We assessed discrimination by the c‐statistic, predicted probability accuracy by the Brier score, and calibration by calibration graphs, and calibration‐intercept and calibration slope. We compared our new model to the updated ACC‐TAVI and IRRMA MPMs on our population.

**Results:**

We included 9144 TAVI‐patients. The observed early mortality was 4.0%. The final MPM had 10 variables, including: critical‐preoperative state, procedure‐acuteness, body surface area, serum creatinine, and diabetes‐mellitus status. The median c‐statistic was 0.69 (interquartile range [IQR] 0.646–0.75). The median Brier score was 0.038 (IQR 0.038–0.040). No signs of miscalibration were observed. The c‐statistic's temporal‐validation was 0.71 (95% confidence intervals 0.64–0.78). Our model outperformed the updated currently available MPMs ACC‐TAVI and IRRMA (*p* value < 0.05).

**Conclusion:**

The new TAVI‐model used additional variables and showed fair discrimination and good calibration. It outperformed the updated currently available TAVI‐models on our population. The model's good calibration benefits preprocedural risk‐assessment and patient counseling.

**Abbreviations**
ACC‐TAVI (ACC TVT): American College of Cardiology Transcatheter Valve Therapy
Amsterdam UMC: Amsterdam University Medical Centres ‐ location AMC (Academic Medical Centre)
AU‐PRC: area under the precision‐recall curve
AU‐ROC: area under the receiver operating‐characteristic curve
BSA: body surface area
BSS: Brier Skill Score
EuroSCORE: European System for Cardiac Operative Risk Evaluation
FRANCE‐2: French Aortic National CoreValve and Edwards^15^
LVEF: left ventricular ejection fraction
MPM: mortality prediction models
NHR: Netherlands Heart Registration (“Nederlandse Hart Registratie” in Dutch)
NYHA: New York Heart Association
TAVI‐MPM: TAVI‐specific MPM
TAVI (TAVR): transcatheter aortic valve implantation (replacement)

## 1 INTRODUCTION

Transcatheter aortic valve implantation (TAVI) was introduced as a treatment of choice for patients suffering from severe symptomatic aortic stenosis who are no candidates for surgical aortic valve replacement (SAVR) or considered to have an increased surgical risk. Heart teams estimate the post‐procedural (30‐day) early mortality risk for heart procedures (including TAVI) using general surgical mortality prediction models (MPM) such as the European System for Cardiac Operative Risk Evaluation (EuroSCORE‐II, 2012),^1^, ^2^ and the Society of Thoracic Surgeons Predicted Risk of Mortality (STS‐PRoM).^3^ These surgical MPMs are known to have poor performance in predicting early mortality on TAVI populations.^4^, ^5^, ^6^ On the other hand, among the currently available TAVI‐specific models (TAVI‐MPMs), the ACC‐TAVI,^7^ and FRANCE‐2^8^ were shown to have, relatively, the best performance when externally validated on TAVI populations. However, this performance is still poor,^4^, ^5^ even when these MPMs are first updated on various external data set.^9^ Therefore, development of a new validated TAVI‐MPM in large registries is important. In this paper, we describe and investigate the performance of a newly developed TAVI‐MPM using the Netherlands Heart Registration (NHR) cohort.

Unlike earlier model‐updates of the currently available TAVI‐MPMs, this new TAVI‐MPM estimates coefficients for each separate variable anew, and considers additional candidate variable predictors like body surface area (BSA), diabetes mellitus (DM) status, serum creatinine, and frailty‐status. The currently available TAVI‐MPMs use different sets of predictors. These predictors include age, gender, left ventricular ejection fraction (LVEF), body mass index (BMI), procedure acuteness status, and TAVI access route (transfemoral and nontransfemoral).^7^, ^8^ None of the currently available TAVI‐MPMs use frailty‐status, DM, or BSA as variable predictors in their models.

Using a comprehensive set of evaluation measures, we perform internal validation, and also temporal (prospective) validation to understand the behavior of the prediction model over time, which best mimics what the model would face in clinical practice.

## 2 METHODS

### 2.1 Study population

In the Netherlands, 16 heart centres submit patients' demographics, clinical characteristics, procedural details mortality status, complications, and follow‐up data for several cardiac procedures to the NHR.^10^ For this study, we obtained the anonymized data on each performed TAVI procedure from the NHR in the period between January 1, 2013 and December 31, 2018. For each patient, to be included in this study, the outcome early mortality should be available. Therefore, we could use data of 13 heart centres out of all 16 heart centres (see flowchart for patients' selection in Figure S1).

### 2.2 Statistical analysis

The primary outcome is postprocedural (30‐day) early mortality, which is defined as death within 30‐days from the TAVI procedures' date. Each center obtained mortality data from the Municipal Personal Records Database.

Candidate variables (predictors) were selected based on the currently available MPMs, reviewing literature, and availability of the predictors in the NHR registry. As candidate predictors, we used patient demographics, past medical history, and procedural details. These predictors were measured and registered before and during any TAVI procedure (Tables 1 and S1). Continuous predictors are presented as mean (with standard deviation [SD]) or median (with interquartile range [IQR]) and were compared between early mortality status groups using the Students' *t* test or the Mann–Whitney test, respectively. Categorical predictors are presented as counts and percentages and were compared using the Chi‐squared test or Fisher exact test as appropriate. A two‐tailed *p*‐value <0.05 was considered statistically significant for all analyses.

**Table 1:** Early (30‐day) mortality rates after TAVI in total population (*n* = 9144), and the univariate analysis of the variable predictors

A. Continuous variable predictors (unit)	Mean (SD)* in nonsurvivors	Univariate analysis* Odds ratio (95% CI), *p* value
Age (years)	80.2 (7.3)	1.01 (0.99–1.03), 0.22
Body surface area (m^2^)	1.83 (0.2)	0.27 (0.16–0.44), <0.001
Left ventricular ejection fraction (%)	47.3 (11.8)	0.98 (0.97–0.98), <0.001
Serum creatinine (μmol/L)	116.7 (69.1)	1.001 (1.00–1.002), 0.004
sPAP (mmHg)	32.9 (12.7)	1.02 (1.01–1.03), 0.001

**B. Categorical variable predictors**	Number of cases (*N*)* ***N* = 9144**	**No of nonsurvivors (*n*) *n* = 368**	**Mortality risk % (*n*/*N* × 100) 4.0%**	**Univariate analysis** * **Odds ratio (95% CI), *p* value**
Gender = Female	4630	190	4.1	1.04 (0.85–1.28), 0.69
Gender = Males	4514	178	3.9	1.0 (ref)
Chronic lung disease (yes)	1970	95	4.8	1.30 (1.02–1.65), 0.03
Chronic lung disease (no)	7139	267	3.7	1.0 (ref)
Extra‐cardiac arteriopathy (yes)	2020	98	4.9	1.32 (1.04–1.67), 0.02
Extra‐cardiac arteriopathy (no)	7073	263	3.7	1.0 (ref)
Neurological dysfunction (yes)	319	11	3.4	0.92 (0.47–1.61), 0.78
Neurological dysfunction (no)	7519	282	3.8	1.0 (ref)
Previous cardiac surgery (yes)	1909	74	3.9	1.01 (0.77–1.30), 0.96
Previous cardiac surgery (no)	6987	269	3.9	1.0 (ref)
Critical preoperative state (yes)	59	15	25.4	8.70 (4.64–15.43), <0.001
Critical preoperative state (no)	8991	339	3.8	1.0 (ref)
Recent myocardial infarction (yes)	168	14	8.3	2.27 (1.25–3.83), 0.004
Recent myocardial infarction (no)	8870	341	3.8	1.0 (ref)
Dialysis (yes)	110	10	9.1	2.47 (1.20–4.53), 0.007
Dialysis (no)	8824	344	3.9	1.0 (ref)
Poor mobility (yes)	565	26	4.6	1.46 (0.94–2.18), 0.08
Poor mobility (no)	5789	185	3.2	1.0 (ref)
CCS class IV angina (yes)	193	8	4.1	1.17 (0.52–2.25), 0.67
CCS class IV angina (No)	6988	249	3.6	1.0 (ref)
Previous CVA (yes)	1009	40	4.0	0.99 (0.70–1.37), 0.96
Previous CVA (no)	8038	321	4.0	1.0 (ref)
Previous aortic valve surgery (yes)	432	26	6.0	1.61 (1.04–2.38), 0.02
Previous aortic valve surgery (no)	8289	317	3.8	1.0 (ref)
Previous permanent pacemaker (yes)	731	27	3.7	0.96 (0.63–1.40), 0.83
Previous permanent pacemaker (no)	7715	297	3.8	1.0 (ref)
Anesthesia (yes)	5457	256	4.7	1.79 (1.40–2.30), <0.001
Anesthesia (no)	3207	86	2.7	1.0 (ref)
Balloon pre‐TAVI (yes)	4090	152	3.7	1.02 (0.81–1.29), 0.85
Balloon pre‐TAVI (no)	4014	146	3.6	1.0 (ref)
PABV (yes)	1127	40	3.5	0.95 (0.67–1.33), 0.79
PABV (no)	6681	248	3.7	1.0 (ref)
Procedure weight (2 operations) (yes)	98	3	3.1	0.78 (0.19–2.09), 0.68
Procedure weight (2 operations) (No)	8627	335	3.9	1.0 (ref)
Procedure urgency				
Elective (ref)	8097	278	3.4	1.0 (ref)
Urgent	805	61	7.6	2.31 (1.72–3.05), <0.001
Emergency	26	6	23.1	8.44 (6.07–19.99), <0.001
Procedure urgency continue variable				2.57 (1.97–3.35), <0.001
Diabetes mellitus (DM) status				
No DM (ref)	6502	267	4.1	1.0 (ref)
DM without medication treatment	394	32	8.1	2.18 (1.46–3.15), <0.001
DM on medication treatment	2056	69	3.3	0.85 (0.65–1.11), 0.25
Functional NYHA class				
NYHA class I and II (ref)	3011	78	2.6	1.0 (ref)
NYHA class III	4472	197	4.4	1.73 (1.33–2.27), <0.001
NYHA class IV	569	47	8.2	3.39 (2.32–4.90), <0.001
Functional NYHA class continue variable				1.81 (1.52–2.17), <0.001
TAVI Access route				
Access route Transfemoral (ref)	7075	232	3.3	1.0 (ref)
Access route subclavian artery	485	23	4.7	1.47 (0.92–2.23), 0.09
Access route Transapical	650	49	7.5	2.40 (1.73–3.28), <0.001
Access route direct aortic	694	49	7.1	2.24 (1.61–3.05), <0.001
Frailty status category	1296	41	N.A.	
Not fragile, category 1 (0–3) (ref)	691	17	2.5	1.0 (ref)
Mild fragile, category 2^4^, ^5^	367	15	4.1	1.80 (0.87–3.70), 0.11
Moderate fragile, category 3^6^, ^7^, ^8^	201	8	4.0	1.75 (0.70–4.04), 0.21
Severe fragile, category 4^9^, ^10^, ^11^, ^12^, ^13^, ^14^	36	1	2.8	1.21 (0.07–6.17), 0.86

Abbreviations: Balloon pre‐TAVI, balloon aortic valvuloplasty prior to date of TAVI; CCS class, Canadian Cardiovascular Society grading of angina pectoris; CVA, cerebrovascular accident; DM, diabetes mellitus; LVEF, left ventricular ejection fraction; NA, not applicable; NYHA, New York Heart Association functional Classification; PABV, percutaneous aortic balloon valvuloplasty (TAVI post‐dilation); Ref, reference in the univariate analysis; sPAP, systolic pulmonary arterial pressure; VSR, poet myocardial infarction ventricular septal rupture.

* Values and frequencies presented in this table were calculated before imputation of the missing data.

For missing values of some potential predictors (Table S2), we assumed that they were missing at random, as we have no a priori reason to consider them otherwise. Therefore, five imputation data sets were generated based on the chain equations (MICE).^11^ In the imputation models for each predictor, we used all other available candidate predictors including, as recommended, the mortality status as covariates. We excluded a predictor that has more than 30% missing values in the primary analysis and performed a sensitivity analysis where this predictor was imputed and included.

### 2.3 Prediction model development strategy

To avoid having a model that overfits the data, we applied a variables selection strategy. Specifically, we used stepwise backward elimination based on the Akaike Information Criterion (AIC).^12^ The development strategy is applied on the whole data set for obtaining the final model, but model validation was applied only on the test portions of the data sets, as described in the next section.

The development strategy goes through four steps. Step One: generate five imputed data sets containing no missing values. Step Two: on each of the five imputed data sets, fit a logistic‐regression model with the outcome and the potential predictors. Predictors were selected with a stepwise backward elimination based on the AIC.^12^ In this step, we fit the model with all continuous and categorical predictors as defined in the NHR registry but we re‐grouped (re‐clustered) some values of three nonbinary categorical variables, as follows: (1) DM status was re‐grouped into two groups: (a) the reference category, which includes “No DM” for patients with no DM, together with “DM on medication treatment” for patients who have DM and are using medication treatment (oral medication and/or insulin); and (b) “DM without medication treatment” for patients with the status of pre‐existing diagnosis of DM that is controlled with diet or life‐style modifications only; (2) NYHA Class I and class II were grouped in one category, and because this variable reflects a patient's functional capacity, and has a monotonous association with mortality, we represented it as a continuous variable; and (3) TAVI access‐route, which was regrouped into “transfemoral,” “subclavian,” “transapical,” and “direct aortic.” We registered the selected predictors from each model on each of the imputed data sets. The predictors that were selected in all five imputed data sets, were considered as the final set of predictors for the prediction strategy. Step Three: refit a logistic‐regression model with the selected final predictors on each imputed data set to predict early mortality. Step Four: obtain the final prediction model by pooling the estimates (*β* coefficients), their 95% confidence intervals (CI) and standard errors (SE), and the odds ratio (with their 95% CI, and *p* value) across the five models using Rubin's rules.^13^

The final model is represented by its linear predictor (LP). The predicted probability is calculated from this LP using this formula:

$\text{Predicted probability}=\;\frac{1}{1+e^{-\text{LP}}}.$

### 2.4 Internal and prospective‐validation

For internal validation, the entire development strategy was evaluated with a stratified 10‐fold cross‐validation. In particular, the whole strategy is repeated on the corresponding training data sets and tested only on the test data sets. For example, in the first cross‐validation fold, 90% of the data set is used for the multiple imputation and for deciding on the pooled model of that fold, then this model is evaluated on the tenth test data sets each consisting of the 10% remaining data set. We use the median to aggregate the performance over the 10 test data sets. The training and test data sets were imputed separately.

To check for possible consequences to population (or calibration) drift, we also performed temporal (prospective) validation. This enables understanding the behavior of the MPM over time, which best mimics what the MPM would face over time in clinical practice. In the prospective validation, we developed a model using the prediction strategy on the data set of 2013–2017. Then we validated the developed MPM on the imputed data set of 2018.

### 2.5 Comparison of the new model with the currently available TAVI models (model update and external validation)

To compare the performance of our new model with the currently available TAVI‐specific MPM, we selected two of them. The first selected model is the ACC‐TAVI model^7^ because in external validation on our population it showed the best relative performance among other models.^5^ Note that although the FRANCE‐2 model also showed a relatively high performance in the Dutch population, it was not selected as we do not have the variable acute pulmonary edema registered in our cohort. Based on the approach used in^9^ for prediction models' update, we updated the ACC‐TAVI model^7^ with our current cohort.

The second selected model, is the model Israeli TAVR Registry Risk Model Accuracy Assessment (IRRMA).^6^ We selected this model as it has very few predictors. Confirm the approaches for models' external validation Martin et al. and Al‐Farra et al.^4^, ^5^ and for models' update in Al‐Farra et al.,^9^ we externally validated and then updated the model IRRMA^6^ with our current cohort.

Consequently, we compared these two updated models' predictive performances with the performance of our new model.

### 2.6 Predictive performance measures

The predictive performance of the model developmental strategy was measured in terms of discrimination with the c‐statistic, which is equal to the area under the receiver‐operating‐characteristic curve (AU‐ROC)^14^; the balance between the positive predictive value and sensitivity with the area under the precision‐recall‐curve (AU‐PRC)^15^, ^16^; accuracy of predictions with the Brier‐score, and the Brier‐skill Score (BSS)^17^; and calibration with calibration graphs, and the calibration‐intercept and calibration‐slope based on the Cox approach.^14^, ^18^ All performance measures were based on the aggregated cross‐validation predictions on the test folds of the five imputed data sets. Details on all these measures appear in File S1.

We also used a forest plot to show the incremental improvement in the AU‐ROC of the main prediction strategy. We also constructed a nomogram^19^ and provided a computer‐based dynamic nomogram graphical interface for the final model, which allows clinicians to easily calculate the linear predictor (LP) and to estimate the TAVI early‐mortality risk for each patient. Details appear in File S1.

### 2.7 Sensitivity analysis

We performed various sensitivity analyses. We allowed the AIC predictor selection to use restricted cubic splines (RCS) for continuous predictors; the categorical variables were also provided without re‐grouping their values; we allowed predictors to be included in the final model if they appeared three or four out of the five imputed data sets.^20^ We also applied the same development strategy but using LASSO instead of the AIC for variable selection. Moreover, we also imputed the whole data set before splitting it to training and testing data sets in the 10‐fold cross‐validation. Finally, we also included the predictor that has a very large proportion of missing values. Details about all these sensitivity analyses appear in File S2.

All analyses in this study were performed in the R statistical environment (R Foundation for Statistical Computing for Windows V. 3.6.1 (http://www.Rproject.org).^21^ Multiple imputation of the data set was completed using the mice package.^11^ We used the package pROC for constructing ROC curves.^22^ The package rms, DynNom (R Shiny)^23^ were used for constructing the nomogram and dynamic nomogram, respectively. The reporting on this model adheres to the TRIPOD checklist for the reporting of multivariable prediction models^24^ (Table S9).

## 3 RESULTS

We included all patients that underwent TAVI (*N* = 9144) in the period between January 1, 2013 and December 31, 2018 from the NHR, after excluding patients with unknown outcome status (*N* = 510) (see flowchart for patients' selection in Figure S1).

The observed early mortality was 4.0% (*n* = 368). Table 1 shows the early‐mortality percentages for the predictor variables. The mean age of nonsurvivors was 80.2 (SD 7.3) years, and 50.6% of the patients were female. Only 9.3% of the procedures were nonelectively performed. Most of the procedures were performed via transfemoral access (79.5%). Table S1 describes the basic characteristics of the total study population (*N* = 9144), stratified by the early‐mortality status after TAVI (survivors = 8776 vs. nonsurvivors = 368), along with the results of the univariate analysis of each of the variable predictors, before as well after missing value imputation.

Early mortality had a significant univariate positive association with lower BSA (mean 1.8 m^2^ non‐survivals vs. 1.9 m^2^ in survivals, *p*‐value < 0.05), lower BMI (mean 26.5 kg/m^2^ vs. 27.3 kg/m^2^, *p*‐value < 0.05), lower LVEF (mean 47.3% vs. 50.5%, *p*‐value < 0.05) and higher critical‐preoperative status (4.2% vs. 0.5%, *p*‐value < 0.05) (Table S1 details the univariate analysis).

Only the predictor frailty‐status has >30.0% missing values. Therefore, this predictor was excluded from the model development analysis. All other predictors with missing values were multiply imputed (in five data sets) based on the assumption that data were missing at random (Table S2).

Two predictors (endocarditis and post‐myocardial infarction ventricular septal rupture) had a very low occurrence in our cohort, and had no association with the outcome. Hence, they were excluded from the model development analysis.

In total, 28 variables were included as potential predictors. The AIC predictor selection (where each predictor appeared in all five imputed data sets) resulted in a final model (referred to as TAVI‐NHR) with 10 predictor variables. Table 2 shows the predictors of the TAVI‐NHR, the *β* coefficients along with their 95% CI, the SE of each of the coefficients, and their odds ratio with 95% CI and *p* values.

**Table 2:** The predictor variables of the NHR‐TAVI model, with the pooled regression coefficients, standard errors of the regression coefficients, the odds ratios and *p* values

Variable predictor	*β* coefficients (95% CI)	SE	Odds ratio (95% CI), *p* value
Intercept	−2.28 (−4.17 to −0.39)	0.97	OR: N.A., 0.02
Age (years)	0.02 (0.01 –0.04)	0.01	1.02 (1.01–1.04), 0.01
Serum creatinine (μmol/L)	0.002 (0.001–0.003)	0.001	1.001 (1.000–1.003), 0.011
Left ventricular ejection fraction (LVEF) (%)	−0.02 (−0.03 to −0.01)	0.01	0.98 (0.97–0.99), <0.001
Body surface area (BSA) (m^2^)	−1.49 (−2.03 to −0.95)	0.28	0.23 (0.13–0.39), <0.001
NYHA class continues (I‐II, III, and IV)	0.36 (0.18–0.55)	0.09	1.44 (1.19–1.73), <0.001
Procedure acuteness (yes)	0.61 (0.30–0.92)	0.15	1.84 (1.36–2.51), <0.001
Chronic lung disease (yes)	0.24 (‐0.01–0.49)	0.13	1.27 (0.99–1.63), 0.06
Critical preoperative state (yes)	1.64 (0.98–2.30)	0.34	5.15 (2.66–9.97), <0.001
Diabetes Mellitus without medication treatment (yes)	0.92 (0.49–1.31)	0.21	2.47 (1.64–3.71), <0.001
TAVI access route transfemoral (reference)			1.00
TAVI access route Subclavian artery (yes)	0.49 (0.05–0.94)	0.23	1.64 (1.05–2.56), 0.03
TAVI access route transapical (yes)	0.90 (0.57–1.22)	0.16	2.45 (1.78–3.39), <0.001
TAVI access route direct aortic (yes)	0.74 (0.41–1.07)	0.17	2.09 (1.51–2.90), <0.001

Abbreviations: CI, confidence interval; N.A., not applicable; NYHA, New York Heart Association functional Classification; OR, odds ratio; SE, standard errors of the regression coefficients.

**Figure 1:**
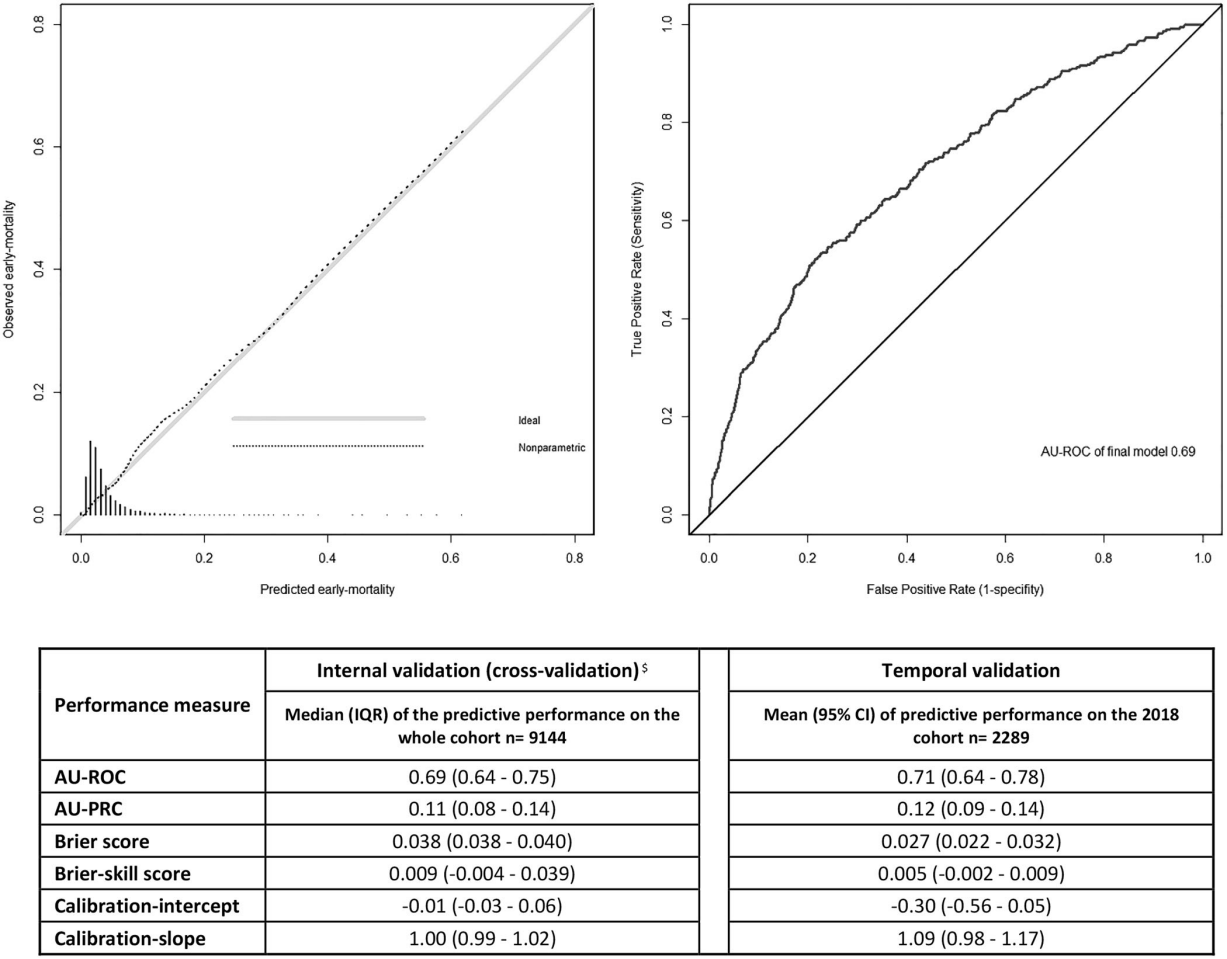
Calibration graph (left) and AU‐ROC (right) of the final model. Performance measures of the internal validation of the prediction strategy in 10‐fold cross‐validation, and performance measures of the temporal validation of the prediction strategy on 2018 data set (*n* = 2289) (below). Abbreviations: AU‐ROC, area under the receiver operating characteristic curve = concordance c‐statistic; AU‐PRC, area under the precision‐recall curve; IQR, interquartile range; 95% CI, 95% confidence intervals. ^$^On each of the training folds, we repeated the prediction strategy (including generating five multiple imputation sets, fitting logistic regression models, selecting variables by stepwise AIC, selecting the variables that appeared five times out of the five imputed data sets, and then pooling the coefficients' estimates for the final model). We tested the final model from each fold on the corresponding test fold. We evaluated the predictive performance in terms of discrimination (AU‐ROC), AU‐PRC, Brier score, Brier‐skill score, calibration intercept, and calibration slope.

In internal validation with the 10‐fold cross‐validation of the prediction strategy, the median AU‐ROC was 0.69 (IQR 0.64–0.75) (Figure 1, right). All models evaluated on the test sets of the cross‐validation were well‐calibrated. The calibration intercepts and calibration slopes did not significantly deviate from their ideal values of 0 and 1, respectively. Figure 1 left, shows the calibration plot of the TAVI‐NHR, with no signs of miscalibration. Figure 1 (lower part) summarizes the performance of the TAVI‐NHR on the test data sets in cross‐validation.

**Figure 2:**
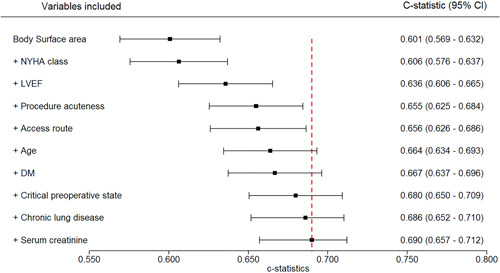
Forest plot showing the AU‐ROC for different models, using different sets of variables from the final selected predictor variables by Akaike Information Criterion (AIC). Abbreviations: NYHA, New York Heart Association functional Classification; LVEF, left ventricular ejection fraction; DM, diabetes mellitus. The estimated early mortality risks derived from these models were evaluated in the test sets. Note that the five imputed data sets were stacked in one data set (where each observation gets a weight of 0.2), and then we split the data set into training and test sets. The variable list shown builds downwards, adding to the existing variables; for example, the c‐statistic (AU‐ROC) shown in the fifth row is for the model that includes body surface area, LVEF, NYHA, procedure acuteness status, and access route. [Color figure can be viewed at wileyonlinelibrary.com]

The forest plot (Figure 2) shows the incremental improvement in the AU‐ROC of the main prediction strategy. The constructed nomogram is presented in Figure S2. Also, a computer‐based dynamic nomogram graphical interface is available online via the link https://nhr-tavi.shinyapps.io/TAVI-NHR/.

In temporal validation, we developed a model on the cohort 2013–2017 (*n* = 6855), and tested it on the 2018‐cohort (*n* = 2289), the AU‐ROC was 0.71 (IQR 0.64–0.78). The model was well‐calibrated with a calibration intercept and calibration slope not significantly deviating from 0 to 1, respectively. Figure 1 (lower part) summarizes the performance of the temporal validation.

The median AU‐ROC of the internally validated AU‐ROC of the updated model ACC‐TAVI in cross‐validation was 0.64 (IQR 0.60–0.73). Detailed results and calibration graph in Table S3 and Figure S3.

The AU‐ROC of the externally validated original IRRMA model was 0.59. The AU‐ROC of the internally validated updated IRRMA was 0.60. Detailed results and calibration graphs appear in Table S4 and Figures S4 and S5.

The predictive performance of our model, in terms of discrimination ability, compared with the performance of these two updated models was statistically significantly higher (*p* value < 0.05).

Sensitivity analysis showed there were no qualitative changes in the results, with one exception. When the variable frailty‐status (86% missing values) was imputed and included as a continuous predictor in the development of a prediction model, it was selected in all five imputed data sets. The median AU‐ROC of this model in cross‐validation was 0.71 (IQR 0.69–0.80). Results of all other sensitivity analyses appear in Supporting Information (File S2, Tables S5, S6, S7A, S7B, and S8A–C).

## 4 DISCUSSION

In this study, we developed a new TAVI‐MPM for early mortality (30 days), based on a large TAVI cohort from the NHR. The TAVI‐NHR includes nine predictors. The TAVI‐NHR had fair discrimination with a median AU‐ROC of 0.69 (IQR 0.64–0.75). The calibration plot shows that the predicted early mortality and the observed early mortality agreed over almost the whole range of probabilities (Figure 1, left). When the predicted probability is between 0.15 and 0.35, the prediction slightly underestimates the proportion of observed early mortality.^25^, ^26^

The model has a statistically significant superior performance to the currently available TAVI‐MPMs even after updating them on our population (*p*‐value < 0.05). Also, in temporal validation the AU‐ROC of TAVI‐NHR was higher than the reported temporally validated AU‐ROC of the updated ACC‐TAVI and FRANCE‐2 in.^9^ The calibration intercept and calibration slope of TAVI‐NHR did not deviate from their ideal values in contrast to the updated models ACC‐TAVI or FRANCE‐2 in.^9^

Most of the currently available TAVI‐MPMs reported original AU‐ROCs in internal validation ranging from 0.59 to 0.66.^6^, ^8^, ^27^ External‐validation studies for TAVI‐MPMs had shown that the currently available models have limited performance on various TAVI populations.^4^, ^5^ In a previously published study, the models ACC‐TAVI and FRANCE‐2 showed the best performance in the Dutch population.^5^ However, this performance was still limited.

In various update studies, these TAVI‐MPMs showed unsatisfactory performance (limited discriminative ability and miscalibration).^9^, ^28^ Unlike the currently available TAVI‐MPMs and their updated versions, the TAVI‐NHR has a higher AU‐ROC and a good calibration in cross‐validation and in temporal validation, and thus better predictions.

Furthermore, in this study, and for comparison, we used our cohort to update the ACC‐TAVI,^7^ and externally validate and update the IRRMA model.^6^ We noted that the performance of TAVI‐NHR is statistically significantly higher than these updated models. Hence, TAVI‐NHR has a superior performance in TAVI‐population, at least in the Netherlands.

The numbers of the used predictors in the currently available TAVI‐MPMs range from four predictors for IRRMA^6^ up to 12 in FRANCE‐2.^8^ The TAVI‐NHR has 10 predictors, which lies in that range. The prediction strategy used in this study has identified three new predictors, which are not used in the currently available MPMs. These are LVEF (%), BSA (m^2^), and DM status (DM without medication treatment).

Several studies^29^, ^30^, ^31^, ^32^, ^33^ have shown that frailty status is a major risk factor for mortality and adding it to MPMs resulted in improved prediction for early mortality. The presence of this vulnerable state has been associated with a high mortality rate in TAVI patients.^34^, ^35^ In our sensitivity analysis, including the imputed frailty‐status, which had a high number of missing values, in the prediction strategy has shown noticeable improvement in the performance in comparison to the TAVI‐NHR. Therefore, our study adds to the current evidence that allowing frailty status as a predictor could improve performance. However, this requires further assessment in large registries. Routinely collecting the frailty status and including it in MPMs has hence the potential to improve models and better aid heart teams in risk identification.

Our study has the following strengths. We used a recent and large national sample of >9000 TAVI patients over 6 years from the NHR. Another strength is that we used AIC with a backward selection of potential predictors thus refraining from the common use of predictor selection based on univariate analysis. Our study reports, in addition to discrimination and calibration, also on AU‐PRC, Brier‐score, and BSS. Finally, we performed a range of sensitivity analyses to gain insight into the stability of the results and findings.

The main limitation of this study may stem from the fact that the analysis is based on a registry of routinely collected data. In such studies, in general, data collection and patients' selection for undergoing the procedure may not be standardized among the different participating centers; there is limited data verification; there are missing data in the centers, and cases are not strictly monitored like in prospective studies and randomized controlled studies. However, the quality of the user data in our current study seems to be reasonable, and it had a few missing values for almost all the used candidate predictors in the model development analysis. Moreover, the NHR requires a standard collection of variables from all the heart centers; the data validity is automatically checked upon upload in the registry, and the NHR performs annual quality checks and audits of the data. Another limitation is that in our cohort of TAVI patients, both old and new generation TAVI‐ prostheses were used. However, this heterogeneous cohort also reflects current clinical practice. Another important limitation of this study is related to the significant proportion of patients with missing values of the variable frailty status. However, we showed in a sensitivity analysis that Including this variable when imputed has improved the predictive performance of the respective model, with a median AU‐ROC in cross‐validation of 0.71 (IQR; 0.69–0.80) (details of the other model and the performance measures appear in Table S8A–C). Multiple imputations should at least partly reflect the variability of this variable, and the use of multiple imputations for such highly missing variables under the assumption of missing at random may be justified.^36^ On the other hand, in daily clinical practices, it seems that this variable were only measured and collected if the patient was at higher risk for surgical aortic valve replacement. We, therefore, recommend that clinicians and national registries measure this variable for each TAVI‐candidate patient.

Our validated MPM may provide useful feedback for heart teams to identify patients who are at high‐risk for early mortality. Our model has a good calibration and may support patient selection and counseling. An implementation of the TAVI‐NHR model as a dynamic nomogram TAVI‐NHR can be used as an easy‐to‐use tool (See Figure S2). This allows users, at least in the Netherlands, to easily calculate the risk of early mortality after TAVI.

We also provided a computer‐based dynamic nomogram graphical interface for the TAVI‐NHR. The resulting dynamic nomogram gives (graphically and numerically) the predicted early‐mortality risk (and the corresponding 95% CI) for any chosen set of values of the independent predictors.

The evaluation of our model on an independent external TAVI population, in other countries, merits future work. Based on this experience the necessity of periodically updating the model should be evaluated and we should obtain a better understanding of calibration drift over time. We also advise researchers to develop prediction models for other outcomes, especially long‐term mortalities after TAVI, and TAVI‐related complications; such as major vascular bleeding, stroke, permanent pacemaker implantation, and renal failure. Finally, it seems that frailty is a promising predictor for early mortality after TAVI, therefore, the impact of this variable on the model should still be confirmed.

## 5 CONCLUSION

Using a large and recent TAVI‐cohort we developed and validated a new TAVI‐MPM with improved discrimination and good calibration. The new model (TAVI‐NHR) outperformed the currently available TAVI‐MPMs and their updated versions. TAVI‐NHR and the provided nomogram implementing the TAVI‐NHR model may be useful for heart teams during patient counseling for risk assessment before the TAVI procedure.

## CONFLICT OF INTEREST

The authors declare no conflict of interest.

## Supporting information

Supplementary information.

Supplementary information.

Supplementary information.

Supplementary information.

Supplementary information.

Supplementary information.

## Data Availability

The data that support the findings of this study are available from the corresponding author upon reasonable request.

## APPENDIX A

The following physicians are members of the NHR THI Registration Committee. They represent the hospitals that have provided the data for this study. Contact with NHR THI Registration Committee can be established via the e‐mail info@nederlandsehartregistratie.nl

M.M. Vis, Amsterdam University Medical Centres.

P. den Heijer, Amphia Hospital.

L. Timmers, St. Antonius Hospital.

W.A.L. Tonino, Catharina Hospital.

C.E. Schotborgh, Haga Hospital.

V. Roolvink, Isala.

F. Porta, Leeuwarden Medical Centre.

M.G. Stoel, Medisch Spectrum Twente.

S. Kats, Maastricht University Medical Centre.

G. Amoroso, Onze Lieve Vrouwe Gasthuis.

H.W. van der Werf, University Medical Centre Groningen.

P.R. Stella, University Medical Centre Utrecht.

N.M.D.A. van Mieghem, Erasmus University Medical Centre.
