# Supplementary figures and images for: Development and validation of a prediction model for early mortality after transcatheter aortic valve implantation (TAVI) based on the Netherlands Heart Registration (NHR): The TAVI‐NHR risk model
Source: Catheter Cardiovasc Interv. 2022 Sep 7;100(5):879–89. doi: 10.1002/ccd.30398 (PMC9826169; doi:10.1002/ccd.30398)

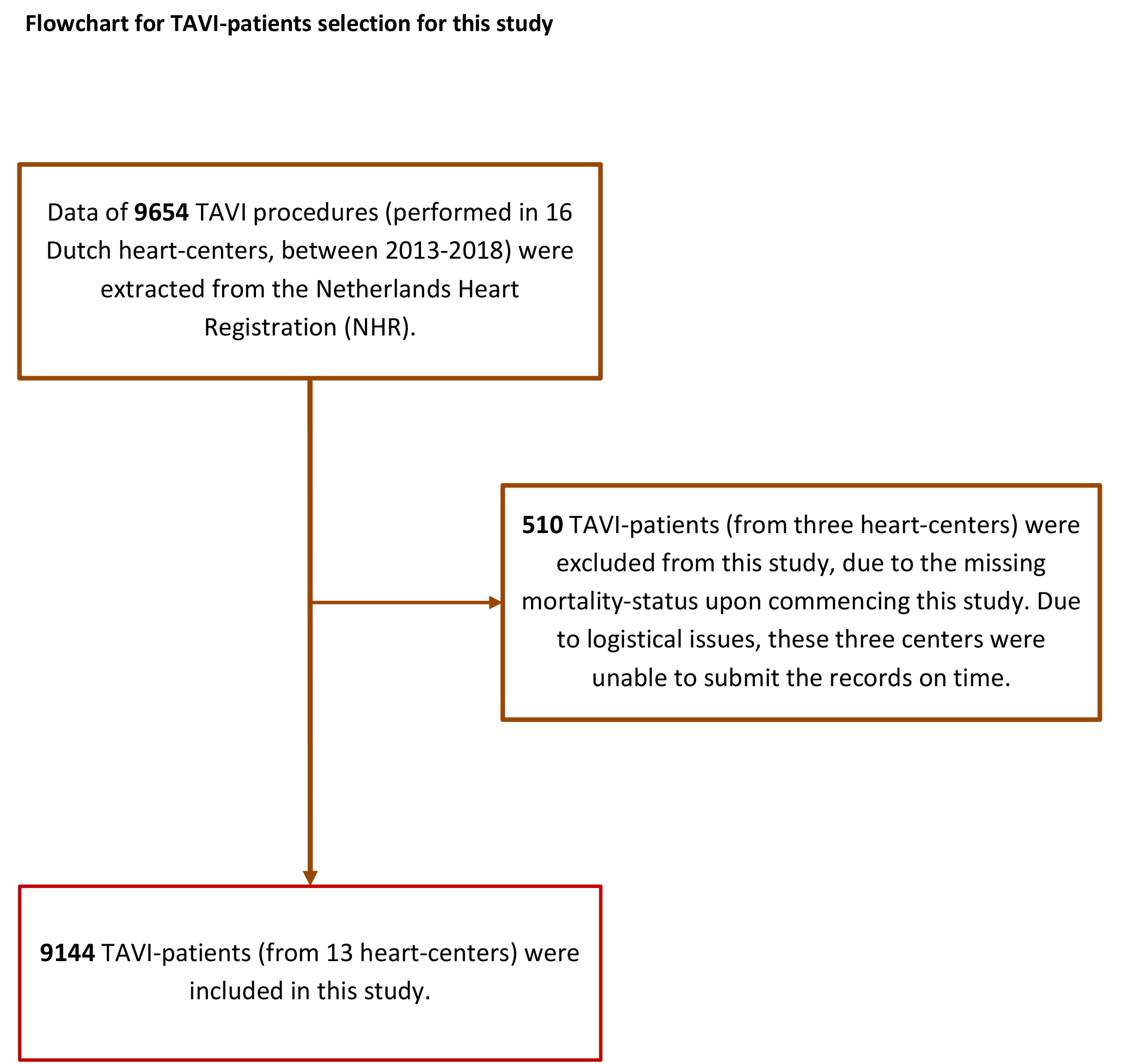

Supplement: Supplementary file 1 — Supplementary information. [file CCD-100-879-s001.tif]

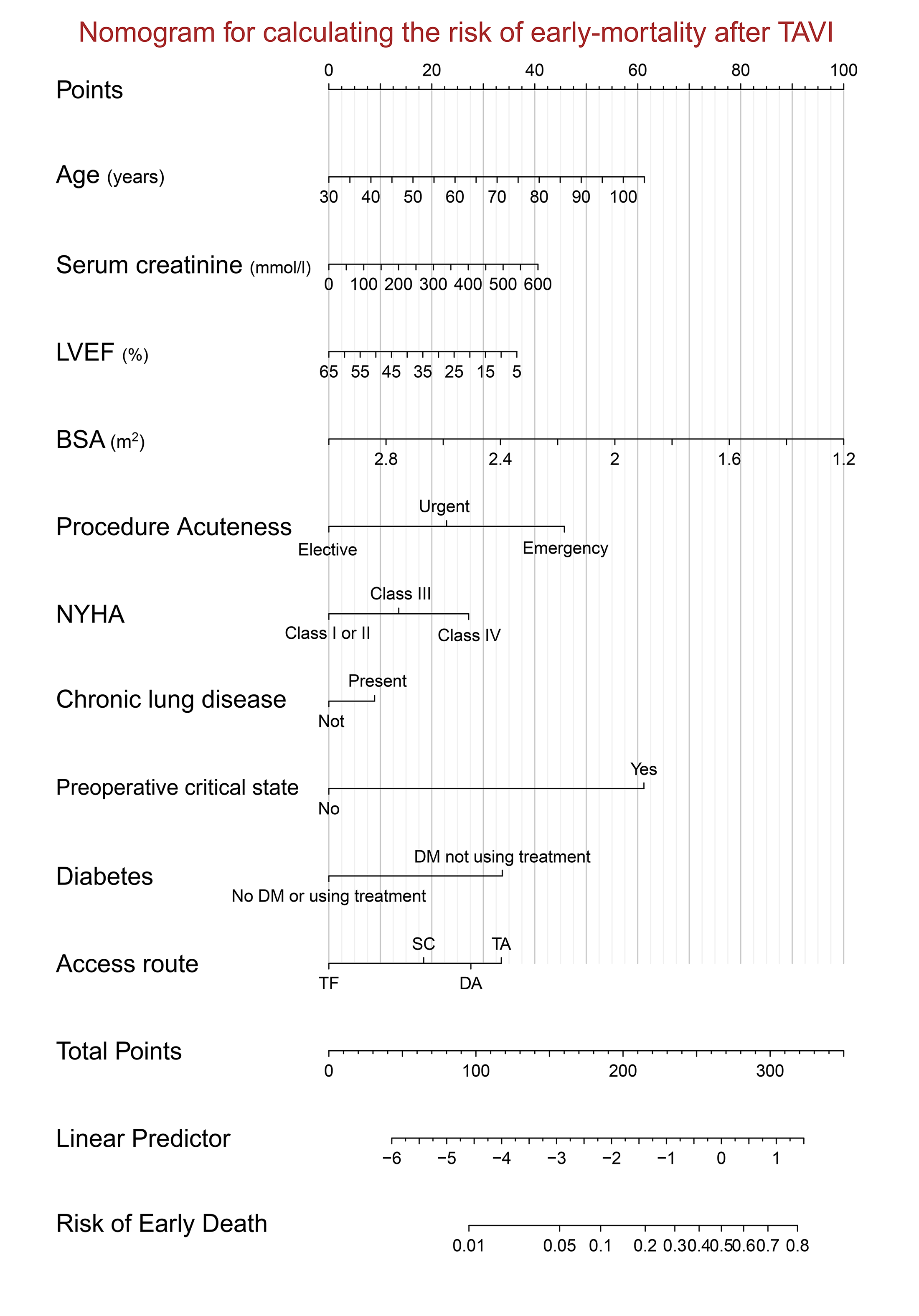

Supplement: Supplementary file 2 — Supplementary information. [file CCD-100-879-s006.tif]

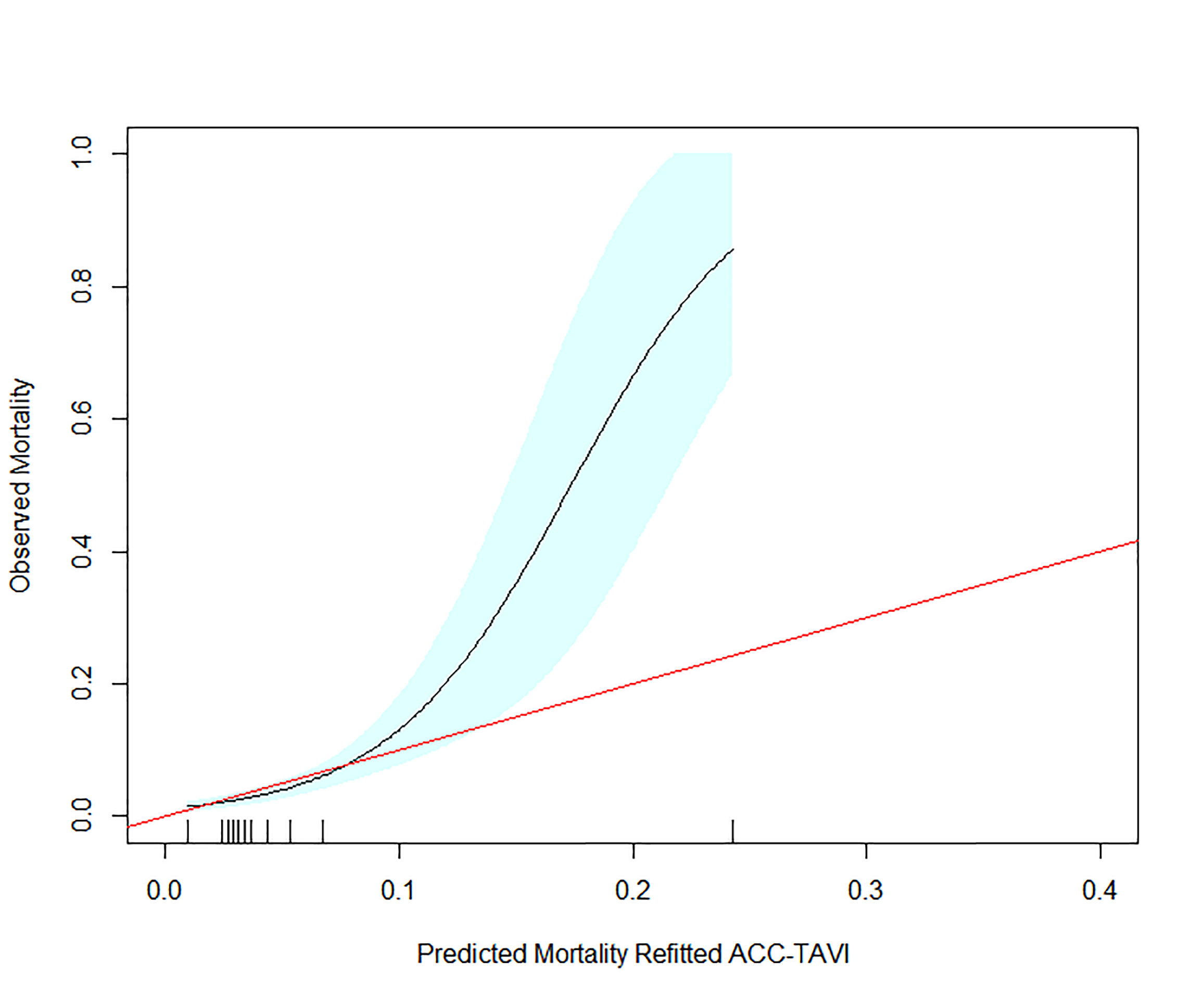

Supplement: Supplementary file 3 — Supplementary information. [file CCD-100-879-s002.tif]

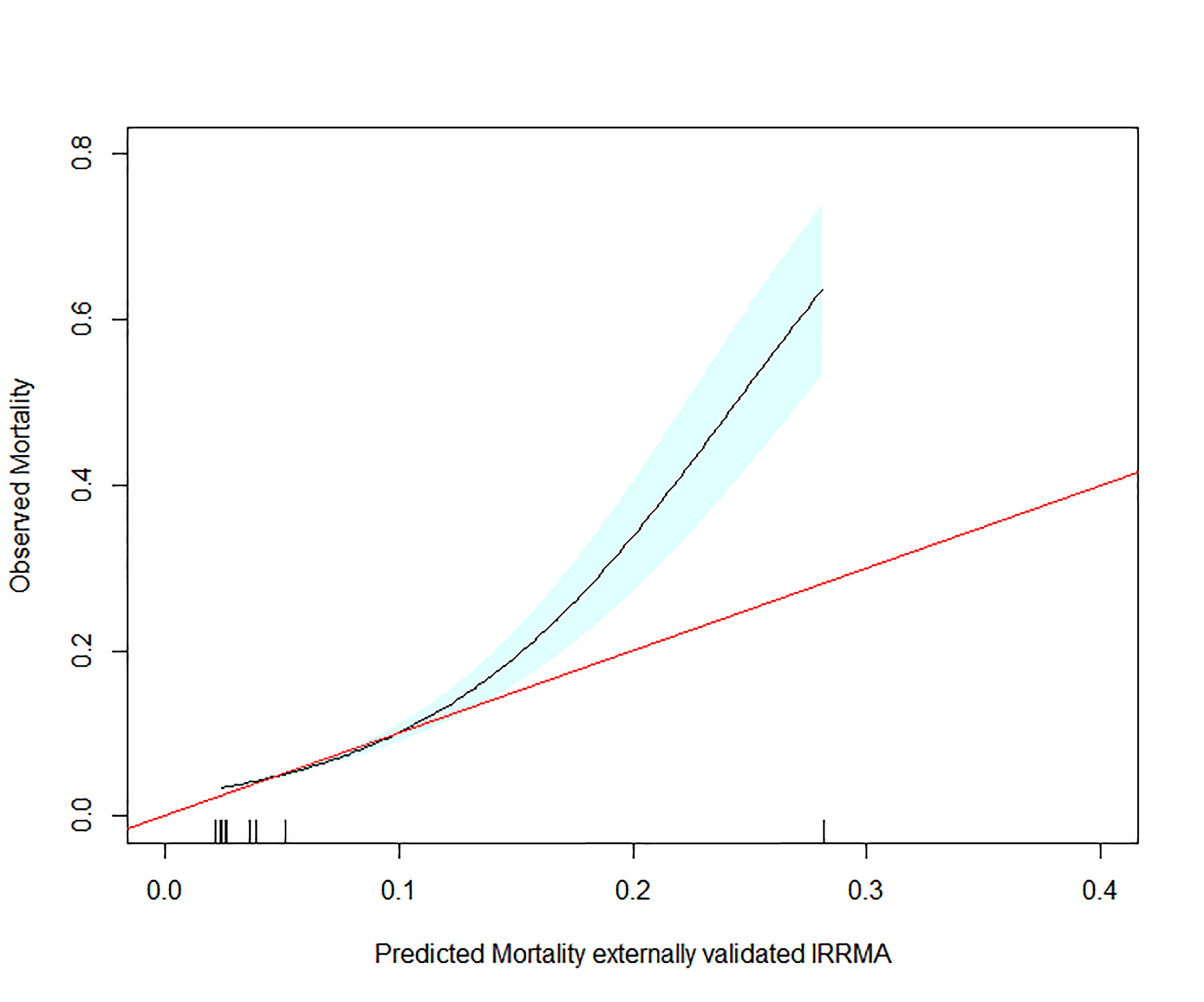

Supplement: Supplementary file 4 — Supplementary information. [file CCD-100-879-s005.tif]

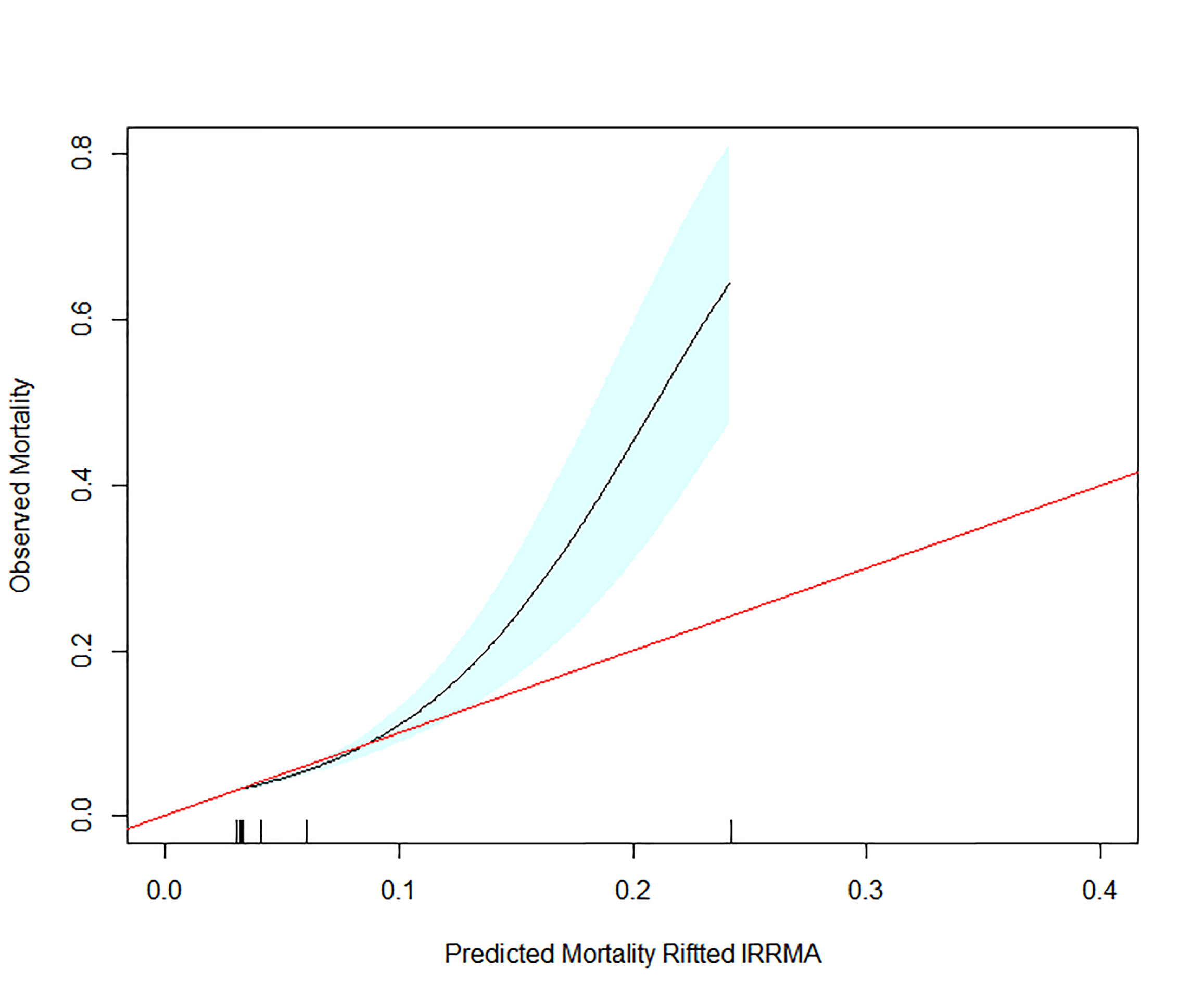

Supplement: Supplementary file 5 — Supplementary information. [file CCD-100-879-s004.tif]
