# Supplementary material for: Development and validation of a prediction model for early mortality after transcatheter aortic valve implantation (TAVI) based on the Netherlands Heart Registration (NHR): The TAVI‐NHR risk model
Source: Catheter Cardiovasc Interv. 2022 Sep 7;100(5):879–89. doi: 10.1002/ccd.30398 (PMC9826169; doi:10.1002/ccd.30398)
Supplement: Supplementary file 6 — Supplementary information. [file CCD-100-879-s003.docx]

**Development and validation of a prediction model for early-mortality after Transcatheter Aortic Valve Implantation (TAVI) based on the Netherlands Heart Registration (NHR): The TAVI-NHR risk model**

**ONLINE-SUPPLEMENTARY MATERIALS**

**CONTENTS:**

**E-supplementary text 1. Definitions of the used predictive performance measures**

**E-supplementary text 2. Details about the performed sensitivity analyses and their results**

**E-supplementary tables**

**E-supplement table 1. Basic characteristics of the total (N=9144) TAVI-population, stratified by the status of the early 30-day mortality after TAVI (survivors = 8776, vs. non-survivors = 368)**

**E-supplementary table 2. Missing values and percentages of the variable predictors before imputation of the NHR TAVI dataset of 9144 patients**

**E-supplementary table 3. Results of the cross-validated predictive performance of model revision ACC-TAVI on TAVI-NHR cohort 2013-2018**

**E-supplementary table 4. Results of the external validation and the performance of the updated version of the Model revision IRRMA on TAVI-NHR cohort 2013-2018**

**E-supplement table 5. Observed early-mortality rates for the continuous variables divided in 3, 4, or 5 subgroups.**

**E-supplementary table 6. Performance measures of the internal validation of the prediction strategy in 10-fold cross-validation, for including predictors appearing in at least 3 or 4 of the 5 imputation datasets**

**E-supplementary table 7A. Predictor variables for the TAVI logistic regression model predicting the 30-day early-mortality, variable selection based on LASSO**

**E-supplementary table 7B. Internal validation of the the prediction strategy based on LASSO**

**E-supplementary table 8A. Predictor variables and their pooled coefficients, odds ratios, standard errors (SE), and p value for the model after including the variable frailty-status in the five imputed datasets**

**E-supplementary table 8B. Predictor variables and their pooled coefficients, odds ratios, standard errors (SE), and p value for the model after including the variable frailty-status in the five imputed datasets from the dataset that include the patients with complete frailty-status score (n = 1295)**

**E-supplementary table 8C. Results of the sensitivity analysis in which we included the variable frailty-status. The performance measures of the internal validation of this prediction strategy in 10-fold cross-validation of these two models**

**E-supplementary table 9. Research Reporting Guideline checklist TRIPOD: Prediction model development and validation**

**E-supplementary figure** *(submitted individually)*

**E-supplementary figure 1. Flow chart for TAVI-patients’ selection for this study**

**E-supplementary figure 2. Nomogram for early-mortality risk calculation after TAVI based on the NHR cohort 2013-2018**

**E-supplementary figure 3. Calibration plot of the updated and refitted ACC-TAVI (model revision) on TAVI-NHR cohort 2013-2018**

**E-supplementary figure 4. Calibration plot of the external validation the model IRRMA on TAVI-NHR cohort 2013-2018**

**E-supplementary figure 5. Calibration plot of the updated and refitted IRRMA (model revision) on TAVI-NHR cohort 2013-2018**

# E-supplementary text 1. Definitions of the used predictive performance measures

**Discrimination (c-statistic), the area under the receiver operating characteristic (AU-ROC)**

Discrimination measures the ability of the MPM to discriminate between patients with the outcome (non-survivals) from those patients without the outcome (survivals). The closer the AU-ROC is to one, the better the discrimination is. We present the ROC curve of the final model and summarize the AU-ROC using the median and over the 10-folds [1].

**The area under the precision-recall-curve (AU-PRC)**

In an imbalanced dataset where the incidence of the outcome is low, the AU-ROC does not provide insight into the balance between the sensitivity and the PPV [2-4]. Therefore, besides the AU-ROC, we also obtain AU-PRC. The AU-PRC shows the trade-off between the precision and the recall of the MPM using different probability thresholds [5]. The terms “recall” and “precision”, correspond respectively to the sensitivity and PPV. The closer the AU-PRC is to one, the better the MPM is.

**Calibration**

Calibration is the degree of agreement between the predicted and observed early-mortality rates across the full probability range. For obtaining the calibration-intercept and calibration-slope according to the Cox approach [6], one fits a logistic regression model for early-mortality using the log-odds of the predictions as the only predictor. If the predicted probabilities were perfectly calibrated, then the coefficients of the linear predictor of this logistic regression model would be 0 for the calibration-intercept and 1 for the calibration-slope. Good calibration is observed if the 95% confidence interval (CI) for the calibration-intercept includes zero, and the 95% CI of the calibration-slope includes one. The calibration graph of the final model (from the AIC variable selection strategy) is also presented, based on the average predictions per patient of the final model on the five imputation datasets.

**Accuracy of predictions**

We measured the accuracy of the predictions with the Brier-score, which is the mean squared error of the predicted early-mortality [7]. A Brier-score ranges from zero to one, the lower the better. For better insight, the Brier-score is converted into the Brier-Skill Score (BSS). The BSS measures the proportional improvement of the predictions over a non-informative reference model that simply predicts the prior probability of the early-mortality for all patients. The maximum value for BSS is 1, which indicates a perfect deterministic prediction, i.e. the model was able to exactly predict the observed outcomes [8]. A BSS of zero means that there is no improvement compared to the predictions of the reference model.

**Forest plot**

We also show the incremental improvement in the AU-ROC of the main prediction strategy via a forest plot. The forest plot shows the improvement in the AU-ROC where the predictors are shown with a decreasing contribution to the AU-ROC. The variable list builds downwards, adding to the existing variables; for example, the AU-ROC shown in the fifth row is for the model that includes NYHA, access route, serum creatinine, LVEF, and body surface area. For this plot, the five imputed datasets were stacked to form one dataset (where each observation gets a weight of 0.2), and then we split a sample for training (75%) and testing (25%), where each patient’s observations are all either in the training or test set. The estimated early-mortality risks derived from these models were evaluated in the test set.

**Nomogram**

Using the approach in [9], we constructed a nomogram to present the final model. A nomogram is a mathematical method or model that is used to predict certain endpoints, such as early-mortality [9], based on several predictors. It is a graphical calculating device that allows clinicians and other users to easily calculate the linear predictor (LP) and the risk of early-mortality after TAVI. By indicating a predictor’s value by a user, the nomogram shows the points associated with this value. The total number of points can then be used to calculate the LP and the associated risk of early-mortality. We also provided a computer-based dynamic nomogram graphical interface for the final model. For this, we used the approach and the materials (R-code) from [10].

# E-supplementary text 2. Details about the performed sensitivity analyses and their results

**1. Methods used for Sensitivity analysis**

**A. Model development with variables selection based on AIC and applying restricted cubic splines (RCS)**

During model development, the continuous (numeric) variables (such as age, BAS, BMI, and LVEF) were kept the same. However, in sensitivity analysis, when the relationship between the continuous variables and the outcome seemed not monotonous, we used the restricted cubic spline (RCS) transformation to assess the effect on variable selection. The RCS was used in step two; all the other steps were kept the same. In step two, we fit the logistic regression model using the RCS for the numeric variables with three or more knots, as necessary, to allow for a non-linear relationship between the variable and the log odds of the outcome.

**B. Model development with variables selection based on AIC and applying grouping and ungrouping of categorical variables**

Unlike the main analysis, we also used categorical variables as they were originally defined in the NHR registration, without regrouping them.

**C. Model development with variable selection based on AIC with applying various majority approaches**

During model development, we checked the effect of also including variables selected by the AIC in at least three or at least four out of the five imputation datasets [11].

**D. Model development with variable selection based on the least absolute shrinkage and selection operator (LASSO)**

To assess the effect of using an alternative approach for variable selection other than the AIC, we performed a sensitivity analysis in which we applied the same model development strategy but with the LASSO instead of the AIC. We determined lambda (a hyperparameter that controls the penalty given to model complexity in terms of the number of included predictors) based on 10 cross-validation. The penalty results in shrinking the model coefficients towards 0 and some variables will have their coefficients shrunk to exactly 0 meaning they are not selected in the model selected final predictor variables.

**E. Dealing with missing values during model validation**

During model validation (internal and temporal-validation) we imputed the training and test sets separately. However, we also performed a sensitivity analysis in which the training and test sets are jointly imputed before being separated. Moreover, we measured the effect of including a variable that has a very large proportion of missing values, on the predictive performance measures.

**2. Results of Sensitivity analysis**

After applying the transformation with RCS on the continuous variables, the predictive performance of these models observed to be inferior to the performance of the final model. In E-supplementary table 2, the categories of the discretized continuous variables are shown with their (monotonously) increasing mortality risk. In the prediction model we used the continuous to avoid information loss.

Fitting the models with the ungrouped categorical variables observed to have lower predictive performance.

Allowing variables that are selected in at least three or four out of five imputation datasets, resulted in models having 14 and 12 variables, respectively. The AU-ROC of these two models were 0.67 and 0.66, respectively. All the predictive performance of these models in cross-validation is given in E-supplementary table 6.

Performing LASSO for variable selection (when including variables that are selected in at least five, four, or three out of five imputation datasets) resulted in models with 17, 22 and 24 variables, respectively. The median AU-ROC in cross-validation of the model when including variables emerging in all five imputation datasets was 0.68 (IQR; 0.65-0.74). E-supplementary table 7A and 7B show the predictor variables (and their coefficients) and details about the predictive performance of these models.

Imputing the whole dataset before dividing to training and testing sets showed no differences from imputing each partition separately.

In the last sensitivity analysis, the variable frailty-status (with 86% missing values) was imputed and included as a continuous variable in the development of a prediction model. The AIC has selected this variable as it appeared in all five imputed datasets. The median AU-ROC in cross-validation was 0.71 (IQR; 0.69-0.80). E-supplementary tables 8A, 8B and 8C provide details about the selected variables and performance of this sensitivity analysis.

# E-supplementary tables

**E-supplement table 1. Basic characteristics of the total (N=9144) TAVI-population, stratified by the status of the early 30-day mortality after TAVI (Survivors = 8776, vs. non-Survivors = 368)**

| **Continuous variable predictors (unit)** | **Overall Mean (SD)** | **Survivors Mean (SD)** | **Non-** **survivors Mean (SD)** | **Test p-value ^*^** |  | **Univariate analysis before imputation Odds ratio (95% CI), P-value** | **Univariate analysis after imputation**  **Odds ratio (95% CI), P-value** |
| --- | --- | --- | --- | --- | --- | --- | --- |
| **Age (years)** | 79.80 (6.90) | 79.78 (6.88) | 80.24 (7.32) | 0.215 |  | 1.01 (0.99 - 1.03), 0.22 | 1.01 (0.99 - 1.03), 0.22 |
| **Body Surface Area (m^2^)** | 1.89 (0.22) | 1.89 (0.22) | 1.83 (0.22) | <0.001 |  | 0.27 (0.16 - 0.44), < 0.001 | 0.26 (0.16 – 0.43), <0.001 |
| **Body Mass Index (kg/m^2^)** | 27.29 (6.00) | 27.32 (6.01) | 26.47 (5.57) | 0.009 |  | 0.97 (0.94 - 0.99), 0.003 | 0.97 (0.94 - 0.99), 0.003 |
| **Serum creatinine (μmol/L)** | 106.82 (65.57) | 106.41 (65.39) | 116.66 (69.05) | 0.004 |  | 1.001 (1.00 - 1.002), 0.004 | 1.002 (1.00 - 1.003), 0.002 |
| **Left Ventricular Ejection Fraction (%)** | 50.08 (10.52) | 50.20 (10.46) | 47.15 (11.48) | <0.001 |  | 0.98 (0.97 - 0.98), < 0.001 | 0.98 (0.97 - 0.99), <0.001 |
| **Systolic Pulmonary Arterial Pressure (mm Hg)** | 30.71 (10.74) | 30.62 (10.65) | 32.92 (12.73) | 0.001 |  | 1.02 (1.01 - 1.03), 0.001 | 1.02 (1.01 - 1.03), 0.01 |
| **Categorical variable predictors** | **Overall (%)^†^**  **N=9144** | **Survivors (%)^±^**  **N=8776** | **Non-** **survivors (%)^φ^**  **N=368** | **Test p-value ^*^** |  | **Univariate analysis before imputation Odds ratio (95% CI), P-value** | **Univariate analysis after imputation**  **Odds ratio (95% CI), P-value** |
| **Male (%)** | 4514 (49.4) | 4336 (49.4) | 178 (48.4) | 0.703 |  | 1.0 (ref) | 1.0 (ref) |
| **Female (%)** | 4630 (50.6) | 4440 (50.6) | 190 (51.6) | 0.736 |  | 1.04 (0.85 - 1.28), 0.69 | 1.04 (0.85 - 1.28), 0.69 |
| **Chronic lung disease (%)** | 1970 (21.6) | 1875 (21.4) | 95 (26.2) | 0.035 |  | 1.30 (1.02 - 1.65), 0.03 | 1.30 (1.02 - 1.64), 0.04 |
| **Extra-cardiac arteriopathy (%)** | 2020 (22.2) | 1922 (22.0) | 98 (27.1) | 0.025 |  | 1.32 (1.04 - 1.67), 0.02 | 1.30 (1.03 - 1.65), 0.03 |
| **Neurological dysfunction (%)** | 319 (4.1) | 308 (4.1) | 11 (3.8) | 0.898 |  | 0.92 (0.47 - 1.61), 0.78 | 0.90 (0.52 - 1.56), 0.70 |
| **Previous cardiac surgery (%)** | 1909 (21.5) | 1835 (21.5) | 74 (21.6) | 1.000 |  | 1.01 (0.77 - 1.30), 0.96 | 1.02 (0.78 - 1.32), 0.90 |
| **Critical preoperative state (%)** | 59 (0.7) | 44 (0.5) | 15 (4.2) | <0.001 |  | 8.70 (4.64 - 15.43), < 0.001 | 9.00 (4.90 - 15.30), < 0.001 |
| **Recent myocardial infarction (%)** | 168 (1.9) | 154 (1.8) | 14 (3.9) | 0.006 |  | 2.27 (1.25 - 3.83), 0.004 | 2.25 (1.29 - 3.92), 0.004 |
| **Dialysis (%)** | 110 (1.2) | 100 (1.2) | 10 (2.8) | 0.011 |  | 2.47 (1.20 - 4.53), 0.007 | 2.35 (1.22- 4.54), 0.01 |
| **Poor mobility (%)** | 565 (8.9) | 539 (8.8) | 26 (12.3) | 0.097 |  | 1.46 (0.94 - 2.18), 0.08 | 1.35 (0.87 - 2.09), 0.17 |
| **CCS class IV angina (%)** | 193 (2.7) | 185 (2.7) | 8 (3.1) | 0.816 |  | 1.17 (0.52 - 2.25), 0.67 | 1.14 (0.46 - 2.87), 0.76 |
| **Previous CVA (%)** | 1009 (11.2) | 969 (11.2) | 40 (11.1) | 1.000 |  | 0.99 (0.70 - 1.37), 0.96 | 0.98 (0.70 - 1.37), 0.92 |
| **Previous aortic valve surgery (%)** | 432 (5.0) | 406 (4.8) | 26 (7.6) | 0.031 |  | 1.61 (1.04 - 2.38), 0.02 | 1.61 (1.05 - 2.47), 0.03 |
| **Previous permanent pacemaker (%)** | 731 (8.7) | 704 (8.7) | 27 (8.3) | 0.913 |  | 0.96 (0.63 - 1.40), 0.83 | 0.98 (0.66 - 1.46), 0.93 |
| **Anesthesia (%)** | 5457 (63.0) | 5201 (62.5) | 256 (74.9) | <0.001 |  | 1.79 (1.40 - 2.30), < 0.001 | 1.78 (1.39 - 2.27), < 0.001 |
| **Balloon pre-TAVI (%)** | 4090 (50.5) | 3938 (50.4) | 152 (51.0) | 0.896 |  | 1.02 (0.81 - 1.29), 0.85 | 0.99 (0.75 - 1.30), 0.93 |
| **PABV (%)** | 1127 (14.4) | 1087 (14.5) | 40 (13.9) | 0.855 |  | 0.95 (0.67 - 1.33), 0.79 | 0.97 (0.70 - 1.34), 0.85 |
| **Procedure weight (2 operations) (%)** | 98 (1.1) | 95 (1.1) | 3 (0.9) | 0.876 |  | 0.78 (0.19 - 2.09), 0.68 | 0.72 (0.23 - 2.30), 0.58 |
| **Unstable angina pectoris (%)** | 17 (0.2) | 17 (0.2) | 0 (0.0) | 0.840 |  | 0 (N.A.), 0.96 | 0 (N.A.), 0.96 |
| **Thoracic aortic surgery (%)** | 8 (0.1) | 8 (0.1) | 0 (0.0) | 1.000 |  | 0 (N.A.), 0.97 | 0 (N.A.), 0.98 |
| **Post-myocardial infarction VSR (%)** | 2 (0.0) | 0 (0.0) | 2 (0.6) | <0.001 |  | 0 (N.A.), 0.95 | 0 (N.A.), 0.94 |
|  |  |  |  |  |  |  |  |
| **Procedure urgency** |  |  |  | <0.001 |  |  |  |
| **Elective (%)** | 8097 (90.7) | 7819 (91.1) | 278 (80.6) |  |  | 1.0 (ref) | 1.0 (ref) |
| **Urgent (%)** | 805 (9.0) | 774 (8.7) | 61 (17.7) |  |  | 2.31 (1.72 – 3.05), < 0.001 | 2.43 (1.80 – 3.28), <0.001 |
| **Emergency (%)** | 26 (0.3) | 20 (0.2) | 6 (0.9) |  |  | 8.44 (6.07 – 19.99), < 0.001 | 8.18 (3.27 – 20.42), <0.001 |
| **Procedure urgency continuous variable** |  |  |  |  |  | 2.44 (1.88 – 3.12), <0.001 | 2.57 (1.97 – 3.35), <0.001 |
| **Diabetes Mellitus (DM) status** |  |  |  | <0.001 |  |  |  |
| **No DM (ref) (%)** | 6502 (72.6) | 6249 (72.6) | 253 (71.5) |  |  | 1.0 (ref) | 1.0 (ref) |
| **DM without medication treatment (%)** | 394 (4.4) | 362 (4.2) | 32 (9.0) |  |  | 2.18 (1.46 - 3.15), < 0.001 | 2.19 (1.49 – 3.23), <0.001 |
| **DM on medication treatment (%)** | 2065 (23.0) | 1996 (23.2) | 69 (19.5) |  |  | 0.85 (0.65 - 1.11), 0.25 | 0.84 (0.64 – 1.10), 0.21 |
| **Functional NYHA class** |  |  |  | <0.001 |  |  |  |
| **NYHA class I (%)** | 896 (11.1) | 871 (11.3) | 25 (7.8) |  |  | 1.0 (ref) | 1.0 (ref) |
| **NYHA class II (%)** | 2115 (26.3) | 2062 (26.7) | 53 (16.5) |  |  | 0.90 (0.56 - 1.47), 0.65 | 0.81 (0.51 – 1.27), 0.36 |
| **NYHA class I and II *(combined)* (%)** | 3011 (37.4) | 2933 (37.9) | 78 (24.2) |  |  | 1.0 (ref) | 1.0 (ref) |
| **NYHA class III (%)** | 4472 (55.5) | 4275 (55.3) | 197 (61.2) |  |  | 1.61 (1.07 - 2.51), 0.03 | 1.49 (0.99 – 2.26), 0.06 |
| **NYHA class IV (%)** | 569 (7.1) | 522 (6.8) | 47 (14.6) |  |  | 3.14 (1.93 - 5.23), < 0.001 | 2.95 (1.85 – 4.72), <0.001 |
| **Functional NYHA class continuous variable** |  |  |  |  |  | 1.82 (1.51 – 2.19), <0.001 | 1.81 (1.52 – 2.17), <0.001 |
| **Access route** |  |  |  | <0.001 |  |  |  |
| **Access route Transfemoral (ref) (%)** | 7075 (79.5) | 6843 (80.0) | 232 (65.7) |  |  | 1.0 (ref) | 1.0 (ref) |
| **Access route subclavian artery (%)** | 485 (5.4) | 462 (5.4) | 23 (6.5) |  |  | 1.47 (0.92 - 2.23), 0.09 | 1.44 (0.93 – 2.23), 0.10 |
| **Access route Transapical (%)** | 650 (7.3) | 601 (7.0) | 49 (13.9) |  |  | 2.40 (1.73 - 3.28), < 0.001 | 2.44 (1.78 – 3.34), < 0.001 |
| **Access route direct aortic (%)** | 694 (7.8) | 645 (7.5) | 49 (13.9) |  |  | 2.24 (1.61 - 3.05), < 0.001 | 2.23 (1.61 – 3.09), <0.001 |
| **Frailty status category^¤^** |  |  |  | 0.369 |  |  |  |
| **Not fragile, category 1 (0-3) (ref) (%)** | 691 (53.4) | 675 (53.8) | 16 (40.0) |  |  | 1.0 (ref) | 1.0 (ref) |
| **Mild fragile, category 2 (4-5) (%)** | 367 (28.3) | 352 (28.0) | 15 (37.5) |  |  | 1.80 (0.87 - 3.70), 0.11 | 1.79 (0.88 – 3.68), 0.11 |
| **Moderate fragile, category 3 (6-8) (%)** | 201 (15.5) | 193 (15.4) | 8 (20.0) |  |  | 1.75 (0.70 - 4.04), 0.21 | 1.74 (0.74 – 4.15), 0.20 |
| **Severe fragile, category 4 (9-14) (%)** | 36 (2.8) | 35 (2.8) | 1 (2.5) |  |  | 1.21 (0.07 - 6.17), 0.86 | 1.21 (0.16 – 9.37), 0.86 |

* Test p-value: continuous predictors were tested using Students’ t-test. While categorical predictors were compared using the chi-squared test or Fisher exact test as appropriate

^†^ Percentage defined as = number of all cases from the variable predictor/total number of all patients (9144) x 100

^±^ Percentage defined as = number of survivors with present variable predictor/total number of survivors patients (8776) x 100

φ Percentage defined as = number of fatalities with present variable predictor/total number of fatalities (368) x 100

^¤^ Numbers presented here are only for the patients with measured frailty score status (before imputation of the missing data).

Abbreviations: Balloon pre-TAVI = Balloon aortic valvuloplasty prior to date of TAVI**;** CCS class = Canadian Cardiovascular Society grading of angina pectoris; CI =confidence interval; CVA = cerebrovascular accident; DM = Diabetes mellitus; N.A. = not applicable; NYHA = New York Heart Association functional Classification; PABV = Percutaneous Aortic Balloon Valvuloplasty (TAVI post-dilation), Ref = reference; VSR = poet myocardial infarction ventricular septal rupture.

**E-supplementary table 2. Missing values and percentages of the variable predictors before imputation of the NHR TAVI dataset of 9144 patients**

|  | **Variable predictors** | **Missing^#^** | **%** |
| --- | --- | --- | --- |
|  | **Frailty status** | 7849 | 85.8 |
|  | **Poor mobility** | 2750 | 30.0 |
|  | **systolic Pulmonary Arterial Pressure** | 2479 | 27.1 |
|  | **Canadian Cardiovascular Society grading of angina pectoris** | 1963 | 21.5 |
|  | **Percutaneous Aortic Balloon Valvuloplasty (TAVI post-dilation) (PABV)** | 1336 | 14.6 |
|  | **Neurological dysfunction** | 1306 | 14.3 |
|  | **Functional New York Heart Association functional Classification** | 1092 | 11.9 |
|  | **Balloon aortic valvuloplasty prior to date of TAVI** | 1040 | 11.4 |
|  | **Previous permanent pacemaker** | 698 | 7.6 |
|  | **Endocarditis ^$^** | 654 | 7.2 |
|  | **Anesthesia** | 480 | 5.3 |
|  | **Previous aortic valve surgery** | 423 | 4.6 |
|  | **Procedure weight (2 operations)** | 419 | 4.6 |
|  | **post-myocardial infarction ventricular septal rupture** | 352 | 3.9 |
|  | **Thoracic aortic surgery ^$^** | 335 | 3.7 |
|  | **Body surface area (m^2^)** | 284 | 3.1 |
|  | **Previous cardiac surgery** | 248 | 2.7 |
|  | **Access route** | 240 | 2.6 |
|  | **Procedure acuteness** | 216 | 2.4 |
|  | **Dialysis** | 210 | 2.3 |
|  | **Unstable angina ^$^** | 197 | 2.2 |
|  | **Left Ventricular Ejection Fraction;** | 195 | 2.1 |
|  | **Diabetes Mellitus** | 183 | 2.0 |
|  | **Recent myocardial infarction;** | 106 | 1.2 |
|  | **Previous cerebrovascular accident** | 97 | 1.1 |
|  | **Critical preoperative state** | 94 | 1.0 |
|  | **Extra-cardiac arteriopathy** | 51 | 0.6 |
|  | **Serum creatinine (μmol/L)** | 35 | 0.4 |
|  | **Chronic lung disease** | 35 | 0.4 |

^$^ Not imputed, as all the registered cases were survival, and no incidence of mortality occurred among non-survivals, so technically the imputation of such variables are not possible.

^#^ After imputation all 27 variables were 0 (%) missing values

**E-supplementary table 3. Results of the cross-validated predictive performance of model revision ACC-TAVI on TAVI-NHR cohort 2013-2018**

| **Performance measure model revision ACC-TAVI** | **Value of the validation measure** | **95% CI** |
| --- | --- | --- |
| **AU-ROC** | 0.64 | 0.61-0.73 |
| **AU-PRC** | 0.09 | 0.064-0.16 |
| **Brier score** | 0.038 | 0.035-0.048 |
| **Brier-skill score** | 0.001 | -0.012-0.026 |
| **Calibration-intercept^#^** | -0.012 | -0.09-0.28 |
| **Calibration-slope^#^** | 1.001 | 0.953-1.105 |

^#^ Calibration-intercepts and -slopes for each model were estimated assuming the slope(s) and intercept(s) equal to one and zero respectively. A satisfactory calibration considered if the 95%CI for the calibration-intercept and-slope included the zero and one, respectively.

Abbreviations: AU-ROC = area under the receiver operating characteristic curve = concordance *(c) statistic*; AU-PRC = area under precision-recall curve; CI =confidence interval.

**E-supplementary table 4. Results of the external validation and the performance of the updated version of the Model revision IRRMA on TAVI-NHR cohort 2013-2018**

| **Performance measure of the external validation IRRMA** | **Value of the validation measure** | **95% CI** |
| --- | --- | --- |
| **AU-ROC** | 0.59 | 0.54-0.62 |
| **AU-PRC** | 0.09 | 0.06-0.11 |
| **Brier score** | 0.038 | 0.033-0.044 |
| **Brier-skill score** | 0.002 | -0.01-0.016 |
| **Calibration-intercept^#^** | -0.019 | -0.09-0.106 |
| **Calibration-slope^#^** | 1.002 | 0.961-1.101 |
|  |  |  |
| **Performance measure of the updated version IRRMA** |  | **95% CI** |
| **AU-ROC** | 0.60 | 0.56-0.63 |
| **AU-PRC** | 0.07 | 0.05-0.09 |
| **Brier score** | 0.034 | 0.031-0.041 |
| **Brier-skill score** | 0.001 | -0.007-0.019 |
| **Calibration-intercept^#^** | -0.019 | -0.09-0.106 |
| **Calibration-slope^#^** | 1.002 | 0.961-1.101 |

^#^ Calibration-intercepts and -slopes for each model were estimated assuming the slope(s) and intercept(s) equal to one and zero respectively. A satisfactory calibration considered if the 95%CI for the calibration-intercept and-slope included the zero and one, respectively.

Abbreviations: AU-ROC = area under the receiver operating characteristic curve = concordance *(c) statistic*; AU-PRC = area under precision-recall curve; CI =confidence interval.

**E-supplement table 5. Observed early-mortality rates for the continuous variables divided in 3, or 4 subgroups**.

| **Continuous variable predictors^*^** | **Total number of cases  in each category (N)** | **Number of non-survivors (n)** | **Mortality risk %**  **(n/N x 100)** |
| --- | --- | --- | --- |
| **Age (years)** |  |  |  |
| **Age <75** | 1845 | 64 | 3.5 |
| **Age 75-80** | 2654 | 97 | 3.7 |
| **Age >80** | 5155 | 207 | 4.0 |
|  |  |  |  |
| **Body Surface Area** |  |  |  |
| **BSA <1.6** | 734 | 50 | 6.8 |
| **BSA 1.6 - 2.0** | 5554 | 220 | 4.0 |
| **BSA >2.0** | 2616 | 81 | 3.1 |
|  |  |  |  |
| **Left Ventricular Ejection Fraction** |  |  |  |
| **LVEF >50** | 5743 | 34 | 0.6 |
| **LVEF 30 - 50** | 2696 | 147 | 5.5 |
| **LVEF <30** | 553 | 172 | 31.1 |
|  |  |  |  |
| **Serum creatinine (μmol/L)** |  |  |  |
| **Less than 200 (μmol/L)** | 8878 | 340 | 3.83 |
| **Between 200 – 400 (μmol/L)** | 267 | 17 | 6.37 |
| **More than 400 (μmol/L)** | 92 | 5 | 5.43 |
|  |  |  |  |
| **Systolic Pulmonary Arterial Pressure** |  |  |  |
| **sPAP > 60 mmHg** | 151 | 9 | 6.0 |
| **sPAP 40-60 mmHg** | 805 | 43 | 5.3 |
| **sPAP 25-40 mmHg** | 1307 | 42 | 3.2 |
| **sPAP ≤25 mmHg** | 4434 | 151 | 3.4 |

* Values and frequencies presented in this table were calculated before imputation of the missing data

Abbreviations: BSA = Body Surface Area; LVEF = Left Ventricular Ejection Fraction; sPAP = systolic Pulmonary Arterial Pressure.

**E-supplementary table 6. Performance measures of the internal validation of the prediction strategy in 10-fold cross-validation, for including predictors appearing in at least 3 or 4 of the 5 imputation datasets**

| **Performance measure** | **Value of the validation measure** | **IQR** |
| --- | --- | --- |
| **Performance measure based on majority of 5 voting out 5** |  |  |
| **Number of selected variables = 9^$^** |  |  |
| **AU-ROC** | 0.68 | 0.66-0.72 |
| **AU-PRC** | 0.11 | 0.08-0.17 |
| **Brier score** | 0.039 | 0.035-0.049 |
| **Brier-skill score** | 0.001 | -0.016-0.045 |
| **Calibration-intercept^#^** | -0.03 | -0.15-0.28 |
| **Calibration-slope^#^** | 1.00 | 0.95-1.15 |
| **Performance measure based on majority of 4 voting out 5** |  |  |
| **Number of selected variables = 12^¥^** |  |  |
| **AU-ROC** | 0.67 | 0.63-0.79 |
| **AU-PRC** | 0.10 | 0.06-0.26 |
| **Brier score** | 0.039 | 0.035-0.052 |
| **Brier-skill score** | -0.004 | -0.031-0.074 |
| **Calibration-intercept^#^** | -0.04 | -0.17-0.29 |
| **Calibration-slope^#^** | 0.99 | 0.95-1.16 |
| **Performance measure based on voting selection of 3 out 5** |  |  |
| **Number of selected variables = 14^¤^** |  |  |
| **AU-ROC** | 0.66 | 0.62-0.74 |
| **AU-PRC** | 0.10 | 0.07-0.16 |
| **Brier score** | 0.038 | 0.035-0.046 |
| **Brier-skill score** | -0.002 | -0.015-0.029 |
| **Calibration-intercept^#^** | -0.04 | -0.15-0.24 |
| **Calibration-slope^#^** | 0.99 | 0.95-1.11 |

^#^ Calibration-intercepts and -slopes for each model were estimated assuming the slope(s) and intercept(s) equal to one and zero respectively. A satisfactory calibration considered if the 95%CI for the calibration-intercept and-slope included the zero and one, respectively.

^$^ Variables appeared in 5 imputation datasets: age (years), left ventricular ejection fraction, body surface area, chronic lung disease, critical preoperative state, NYHA class: (class II, class III, and class IV), procedure acuteness: (urgent, and emergency), TAVI access route: (subclavian artery, transapical, and direct aortic), and diabetes mellitus status: (diabetes without treatment and Diabetes on treatment).

**^¥^** Variables appeared in 4 imputation datasets: the above 9 variables, serum creatinine, anesthesia, and systolic pulmonary arterial pressure.

**^¤^** Variables appeared in 3 imputation datasets: the above 12 variables, recent MI and poor mobility.

Abbreviations: AU-ROC = area under the receiver operating characteristic curve = concordance (c) statistic; AU-PRC = area under precision-recall curve; IQR =

Interquartile range

**E-supplementary table 7A. Predictor variables for the TAVI logistic regression model predicting the 30-day early-mortality, variable selection based on LASSO**

| **Variable predictors** | **Coefficients** |
| --- | --- |
| **Intercept** | -2.66 |
| **Age (years)** | 0.02 |
| **Body surface area (BSA) (m^2^)** | -1.09 |
| **Serum creatinine (μmol/L)** | 0.00 |
| **Left Ventricular Ejection Fraction (LVEF) (%)** | -0.01 |
| **sPAP (mm Hg)** | 0.01 |
| **Female (Yes)** | -0.07 |
| **Diabetes mellitus status** |  |
| **Diabetes without treatment medication (Yes)** | 0.72 |
| **Diabetes on treatment medication (Yes)** | -0.12 |
| **Chronic lung disease (Yes)** | 0.17 |
| **Previous cardiac surgery (Yes)** | -0.10 |
| **Critical preoperative state (Yes)** | 1.49 |
| **Unstable angina pectoris (Yes)** | -1.19 |
| **Recent myocardial infarction (Yes)** | 0.39 |
| **Thoracic aortic surgery (Yes)** | -0.28 |
| **Dialysis (Yes)** | 0.28 |
| **Poor mobility (Yes)** | 0.21 |
| **NYHA** |  |
| **NYHA class II (Yes)** | -0.20 |
| **NYHA class III (Yes)** | 0.20 |
| **NYHA class IV (Yes)** | 0.45 |
| **CCS class IV angina (Yes)** | -0.20 |
| **Procedure urgency** |  |
| **Procedure urgency Urgent (Yes)** | 0.53 |
| **Procedure urgency Emergency (Yes)** | 1.08 |
| **Procedure weight** |  |
| **Procedure weight (1 operation) (ref)** |  |
| **Procedure weight (2 operation) (Yes)** | -0.34 |
| **Previous aortic valve surgery (Yes)** | 0.28 |
| **Previous permanent pacemaker (Yes)** | -0.12 |
| **Anesthesia (Yes)** | 0.24 |
| **TAVI access route** |  |
| **TAVI Access route, Subclavian artery (Yes)** | 0.23 |
| **TAVI Access route ,Transapical (Yes)** | 0.65 |
| **TAVI Access route, Direct aortic (Yes)** | 0.53 |
| **Neurological dysfunction (Yes)** | -0.14 |

Abbreviations: CCS class = Canadian Cardiovascular Society grading of angina pectoris; NYHA = New York Heart Association functional Classification; sPAP = systolic Pulmonary Arterial Pressure

**E-supplementary table 7B. Internal validation of the the prediction strategy based on LASSO**

| **Performance measure** | **Value of the validation measure** | **IQR** |
| --- | --- | --- |
| **Performance measure based on voting selection of 5 out 5** |  |  |
| **Number of selected variables = 17^$^** |  |  |
| **AU-ROC** | 0.68 | 0.65-0.74 |
| **AU-PRC** | 0.11 | 0.08-0.18 |
| **Brier score** | 0.038 | 0.034-0.05 |
| **Brier-skill score** | 0.006 | -0.009-0.039 |
| **Calibration-intercept^#^** | -0.01 | -0.13-0.31 |
| **Calibration-slope^#^** | 1.00 | 0.94-1.11 |
| **Performance measure based on majority of 4 voting out 5** |  |  |
| **Number of selected variables = 22^¥^** |  |  |
| **AU-ROC** | 068 | 0.61-0.84 |
| **AU-PRC** | 0.16 | 0.09-0.28 |
| **Brier score** | 0.059 | 0.038-0.083 |
| **Brier-skill score** | -0.012 | -0.023-0.076 |
| **Calibration-intercept^#^** | 0.32 | 0.01-1.27 |
| **Calibration-slope^#^** | 0.88 | 0.79-1.09 |
| **Performance measure based on majority of 3 voting out 5** |  |  |
| **Number of selected variables = 24^¤^** |  |  |
| **AU-ROC** | 0.67 | 0.64-0.76 |
| **AU-PRC** | 0.11 | 0.08-0.18 |
| **Brier score** | 0.039 | 0.037-0.05 |
| **Brier-skill score** | -0.003 | -0.02-0.03 |
| **Calibration-intercept^#^** | -0.02 | -0.16-0.49 |
| **Calibration-slope^#^** | 1.00 | 0.96-1.13 |

^#^ Calibration-intercepts and -slopes for each model were estimated assuming the slope(s) and intercept(s) equal to one and zero respectively. A satisfactory calibration considered if the 95%CI for the calibration-intercept and-slope included the zero and one, respectively.

^$^ Variables appeared in 5 imputation datasets: age (years), body surface area, serum creatinine, left ventricular ejection fraction, female gender, diabetes mellitus status: (diabetes without treatment and Diabetes on treatment), chronic lung disease, critical preoperative state, recent myocardial infarction, poor mobility, NYHA class: (class II, class III, and class IV), procedure acuteness: (urgent, and emergency), procedure weight, previous aortic valve surgery, anesthesia, TAVI access route: (subclavian artery, transapical, and direct aortic), and previous permanent pacemaker.

**^¥^** Variables appeared in 4 imputation datasets: the above 17 variables, systolic pulmonary arterial pressure, previous cardiac surgery, unstable angina pectoris, thoracic aortic surgery, CCS class IV angina,

**^¤^** Variables appeared in 3 imputation datasets: the above 22 variables, dialysis and neurological dysfunction.

Abbreviations: AU-ROC = area under the receiver operating characteristic curve = concordance (c) statistic; AU-PRC = area under precision-recall curve; IQR =

Interquartile range

**E-supplementary table 8A. Predictor variables and their pooled coefficients, odds ratios, standard errors (SE), and p value for the model after including the variable frailty-status in the five imputed datasets**

| **Variable predictor** | **Coefficients (95% CI)** | | | **SE** |  | **OR**  (**95% CI) P-value** | | | |
| --- | --- | --- | --- | --- | --- | --- | --- | --- | --- |
| **Intercept** | -2.64 (-4.69 - -0.59) | | | 0.55 |  | 1.04 (0.07 - 0.01) 0.012 | | | |
| **Age (years)** | 0.02 (0.00 - 0.03) | | | 1.03 |  | 0.01 (1.02 - 1.00) 0.073 | | | |
| **Body surface area (BSA) (m^2^)** | -1.17 (-1.74 - -0.60) | | | 0.55 |  | 0.29 (0.31 - 0.18) 0.000 | | | |
| **Left Ventricular Ejection Fraction (LVEF) (%)** | -0.02 (-0.03 - -0.01) | | | 0.99 |  | 0.01 (0.98 - 0.97) 0.002 | | | |
| **Critical preoperative state (Yes)** | 1.95 (1.25 - 2.65) | | | 14.12 |  | 0.36 (7.02 - 3.49) 0.000 | | | |
| **Procedure acuteness** |  |  |  |  |  |  |  |  |  |
| **Procedure acuteness, Urgent (Yes)** | 0.48 (0.15 - 0.82) | | | 2.27 |  | 0.17 (1.62 - 1.16) 0.005 | | | |
| **Procedure acuteness, Emergency (Yes)** | 1.58 (-0.13 - 3.29) | | | 26.92 |  | 0.79 (4.86 - 0.88) 0.068 | | | |
| **Frailty status score** | 0.19 (0.07 - 0.30) | | | 1.36 |  | 0.05 (1.21 - 1.07) 0.007 | | | |
| **Diabetes mellitus status** |  |  |  |  |  |  |  |  |  |
| **Diabetes without treatment medication (Yes)** | 1.01 (0.56 - 1.46) | | | 4.33 |  | 0.23 (2.75 - 1.75) 0.000 | | | |
| **Diabetes on treatment medication (Yes)** | -0.23 (-0.55 - 0.09) | | | 1.09 |  | 0.16 (0.79 - 0.58) 0.154 | | | |
| **TAVI access route** |  |  |  |  |  |  |  |  |  |
| **TAVI Access route, Subclavian artery (Yes)** | 0.47 (-0.03 - 0.97) | | | 2.65 |  | 0.25 (1.60 - 0.97) 0.066 | | | |
| **TAVI Access route ,Transapical (Yes)** | 0.88 (0.49 - 1.28) | | | 3.59 |  | 0.20 (2.42 - 1.63) 0.000 | | | |
| **TAVI Access route, Direct aortic (Yes)** | 0.76 (0.40 - 1.12) | | | 3.07 |  | 0.18 (2.14 - 1.49) 0.000 | | | |

Abbreviations: CI =confidence interval; OR = odds ratios, SE = standard errors.

**E-supplementary table 8B. Predictor variables and their pooled coefficients, odds ratios, standard errors (SE), and p value for the model after including the variable frailty-status in the five imputed datasets from the dataset that include the patients with complete frailty-status score (n = 1295)**

| **Variable predictor** | **Coefficients (95% CI)** | | | **SE** |  | **OR**  (**95% CI) P-value** | | | |
| --- | --- | --- | --- | --- | --- | --- | --- | --- | --- |
| **Intercept** | -0.76 (-2.10 - 0.58) | | | 1.78 |  | 0.67 (0.47 - 0.12) 0.260 | | | |
| **Left Ventricular Ejection Fraction (LVEF) (%)** | -0.02 (-0.03 - -0.01) | | | 0.99 |  | 0.01 (0.98 - 0.97) 0.001 | | | |
| **Critical preoperative state (Yes)** | 2.24 (1.56 - 2.92) | | | 18.46 |  | 0.34 (9.38 - 4.76) 0.000 | | | |
| **Recent myocardial infarction (Yes)** | 0.28 (-0.39 - 0.95) | | | 2.58 |  | 0.34 (1.32 - 0.68) 0.410 | | | |
| **CCS grade IV (Yes)** | -0.09 (-1.19 - 1.00) | | | 2.72 |  | 0.50 (0.91 - 0.31) 0.855 | | | |
| **Frailty status score** | 0.20 (0.09 - 0.31) | | | 1.37 |  | 0.05 (1.23 - 1.10) 0.003 | | | |
| **Body surface area (BSA) (m^2^)** | -1.34 (-1.88 - -0.80) | | | 0.45 |  | 0.28 (0.26 - 0.15) 0.000 | | | |
| **Diabetes mellitus status** |  |  |  |  |  |  |  |  |  |
| **Diabetes without treatment medication (Yes)** | 0.96 (0.50 - 1.43) | | | 4.17 |  | 0.23 (2.62 - 1.65) 0.000 | | | |
| **Diabetes on treatment medication (Yes)** | -0.20 (-0.51 - 0.11) | | | 1.12 |  | 0.16 (0.82 - 0.60) 0.202 | | | |

Abbreviations: CCS class = Canadian Cardiovascular Society grading of angina pectoris; CI =confidence interval; OR = odds ratios, SE = standard errors.

**E-supplementary table 8C. Results of the sensitivity analysis in which we included the variable frailty-status. The performance measures of the internal validation of this prediction strategy in 10-fold cross-validation of these two models**

| **Performance measure on the 5 imputed**  **datasets including frailty score** | **Value of the validation measure** | **IQR** |
| --- | --- | --- |
| **Number of selected variables = 8** |  |  |
| **AU-ROC** | 0.71 | 0.69-0.80 |
| **AU-PRC** | 0.11 | 0.08-0.17 |
| **Brier score** | 0.039 | 9.034-0.046 |
| **Brier-skill score** | -0.002 | -0.019-0.035 |
| **Calibration-intercept^#^** | -0.02 | -0.14-0.26 |
| **Calibration-slope^#^** | 0.99 | 0.96-1.09 |
| **Performance measure on the**  **datasets with measurement frailty score n = 1295** |  | **IQR** |
| **Number of selected variables = 7** |  |  |
| **AU-ROC** | 0.77 | 0.66-0.91 |
| **AU-PRC** | 0.32 | 0.22-0.58 |
| **Brier score** | 0.033 | 0.021-0.064 |
| **Brier-skill score** | 0.13 | 0.07-0.30 |
| **Calibration-intercept#** | 0.04 | -0.21-0.10 |
| **Calibration-slope#** | 1.00 | 0.81-1.44 |

On each of the inner-folds, we repeated the prediction strategy (including generating five multiple imputation sets, fitting generalized logistic regression models, variable selection by stepwise Akaike information criterion (AIC), applying the majority voting, then pooling the estimate coefficients for the final model). The final model from each fold is tested on each of the outer-folds. The predictive performance was assessed in terms of discrimination, AU-PRC, Brier score, Brier skill score, calibration-intercept and calibration-slope.

^#^ Calibration-intercepts and -slopes for each model were estimated assuming the slope(s) and intercept(s) equal to one and zero respectively. A satisfactory calibration considered if the 95%CI for the calibration-intercept and-slope included the zero and one, respectively.

Abbreviations: AU-ROC = area under the receiver operating characteristic curve = concordance *(c) statistic*; AU-PRC = area under precision-recall curve; IQR=Interquartile range.

**E-supplementary table 9. Research Reporting Guideline checklist TRIPOD: Prediction model development and validation**

| **Section/Topic** | **Item** | **Checklist Item** | **Page** |
| --- | --- | --- | --- |
| **Title and abstract** | | | |
| Title | 1 | Identify the study as developing and/or validating a multivariable prediction model, the target population, and the outcome to be predicted. | 1* |
| Abstract | 2 | Provide a summary of objectives, study design, setting, participants, sample size, predictors, outcome, statistical analysis, results, and conclusions. | 2 |
| **Introduction** | | | |
| Background and objectives | 3a | Explain the medical context (including whether diagnostic or prognostic) and **rationale** for developing or validating the multivariable prediction model, including references to existing models. | 3 |
|  | 3b | Specify the objectives, including whether the study describes the development or validation of the model or both. | 3 |
| **Methods** | | | |
| Source of data | 4a | Describe the study design or source of data (e.g., randomized trial, cohort, or registry data), separately for the development and validation data sets, if applicable. | ✓ |
|  | 4b | Specify the key study dates, including start of accrual; end of accrual; and, if applicable, end of follow-up. | ✓ |
| Participants | 5a | Specify key elements of the study setting (e.g., primary care, secondary care, general population) including number and location of centres. | ✓ |
|  | 5b | Describe eligibility criteria for participants. | ✓ |
|  | 5c | Give details of treatments received, if relevant. | NA |
| Outcome | 6a | Clearly define the outcome that is predicted by the prediction model, including how and when assessed. | ✓ |
|  | 6b | Report any actions to blind assessment of the outcome to be predicted. | ✓ |
| Predictors | 7a | Clearly define all predictors used in developing or validating the multivariable prediction model, including how and when they were measured. | ✓ |
|  | 7b | Report any actions to blind assessment of predictors for the outcome and other predictors. | ✓ |
| Sample size | 8 | Explain how the study size was arrived at. | ✓ |
| Missing data | 9 | Describe how missing data were handled (e.g., complete-case analysis, single imputation, multiple imputation) with details of any imputation method. | ✓ |
| Statistical analysis methods | 10c | For validation, describe how the predictions were calculated. | ✓ |
|  | 10d | Specify all measures used to assess model performance and, if relevant, to compare multiple models. | ✓ |
|  | 10e | Describe any model updating (e.g., recalibration) arising from the validation, if done. | ✓ |
| Risk groups | 11 | Provide details on how risk groups were created, if done. | ✓ |
| Development vs. validation | 12 | For validation, identify any differences from the development data in setting, eligibility criteria, outcome, and predictors. | ✓ |
| **Results** | | | |
| Participants | 13a | Describe the flow of participants through the study, including the number of participants with and without the outcome and, if applicable, a summary of the follow-up time. A diagram may be helpful. | ✓ |
|  | 13b | Describe the characteristics of the participants (basic demographics, clinical features, available predictors), including the number of participants with missing data for predictors and outcome. | ✓ |
|  | 13c | For validation, show a comparison with the development data of the distribution of important variables (demographics, predictors and outcome). | ✓ |
| Model performance | 16 | Report performance measures (with CIs) for the prediction model. | ✓ |
| Model-updating | 17 | If done, report the results from any model updating (i.e., model specification, model performance). | ✓ |
| **Discussion** | | | |
| Limitations | 18 | Discuss any limitations of the study (such as nonrepresentative sample, few events per predictor, missing data). | ✓ |
| Interpretation | 19a | For validation, discuss the results with reference to performance in the development data, and any other validation data. | ✓ |
|  | 19b | Give an overall interpretation of the results, considering objectives, limitations, results from similar studies, and other relevant evidence. | ✓ |
| Implications | 20 | Discuss the potential clinical use of the model and implications for future research. | ✓ |
| **Other information** | | | |
| Supplementary information | 21 | Provide information about the availability of supplementary resources, such as study protocol, Web calculator, and data sets. | ✓ |
| Funding | 22 | Give the source of funding and the role of the funders for the present study. | ✓ |

# REFERENCES

1. Hanley JA, McNeil BJ. The meaning and use of the area under a receiver operating characteristic (ROC) curve. Radiology. 1982;143(1):29-36.
2. Saito T, Rehmsmeier M. The precision-recall plot is more informative than the ROC plot when evaluating binary classifiers on imbalanced datasets. PLoS One. 2015;10(3):e0118432.
3. Ozenne B, Subtil F, Maucort-Boulch D. The precision--recall curve overcame the optimism of the receiver operating characteristic curve in rare diseases. J Clin Epidemiol. 2015;68(8):855-9.
4. Davis J, Goadrich M. The relationship between Precision-Recall and ROC curves. Proceedings of the 23rd international conference on Machine learning; Pittsburgh, Pennsylvania, USA. 1143874:

ACM; 2006. p. 233-40.

1. Boyd K, Eng KH, Page CD, editors. Area under the Precision-Recall Curve: Point Estimates and Confidence Intervals2013; Berlin, Heidelberg: Springer Berlin Heidelberg.
2. Cox DR. Two further applications of a model for binary regression. Oxford University Press on behalf of Biometrika Trust. 1958;45:562-5 (4 pages).
3. Brier G. Verification of Forecasts Expressed in Terms of Probability. Monthly Weather Review. 1950;78:1-3.
4. Murphy AH. A New Vector Partition of the Probability Score. . J Appl Meteorol, National Center for Atmospheric Research, Boulder, Colo. 1973:595-600.
5. Iasonos A, Schrag D, Raj GV, Panageas KS. How to build and interpret a nomogram for cancer prognosis. J Clin Oncol. 2008;26(8):1364-70.
6. Jalali A, Alvarez-Iglesias A, Roshan D, Newell J. Visualising statistical models using dynamic nomograms. PLoS One. 2019;14(11):e0225253.
7. Rubin DB. Multiple Imputation for Nonresponse in Surveys. . New York: John Wiley and Sons; 1987.
